# Fe^3+^-Mediated Interfacial Polyphenol Coatings on Hair Fibers Using an *Aronia melanocarpa* Extract

**DOI:** 10.3390/plants15162500

**Published:** 2026-08-18

**Authors:** Su Yan, Yinghui Gu, Yifei Kong, Yudi Xiang, Bohan Yang, Xiaomeng Su, Li Sheng, Kai Song

**Affiliations:** 1 School of Life Science, Changchun Normal University, Changchun 130032, China; yansu@ccsfu.edu.cn (S.Y.); qx202510003@stu.ccsfu.edu.cn (Y.G.); k18043041919@163.com (Y.K.); 13872530804@163.com (Y.X.); 13756144861@163.com (B.Y.); jixuezcn@163.com (X.S.); 2 Jilin Qiuzhiyuan Ecological Technology Co., Ltd., Lishu County, Siping 136000, China; qzy_shengli@163.com; 3 Institute of Innovation Science and Technology, Changchun Normal University, Changchun 130032, China

**Keywords:** *Aronia melanocarpa*, polyphenol extract, procyanidins, Fe^3+^–polyphenol association, interfacial polyphenol coating, plant-derived hair dyeing

## Abstract

*Aronia melanocarpa* contains abundant phenolic compounds, including procyanidins, flavonols, flavan-3-ols, and phenolic acids, but its pronounced astringency limits its broader use in food applications. Catechol and other oxygen-donor motifs in plant phenolics are known to associate with Fe^3+^ and can support metal–phenolic assembly. In this study, ultrasound-assisted cellulase–pectinase extraction was used to recover an *A. melanocarpa* polyphenol extract, which was subsequently combined with Fe^3+^ to construct a plant-derived hair dye coating on keratin fibers. Under optimized extraction conditions, the total phenolic yield reached 82.315 mg gallic acid equivalents (GAE)/g dry fruit. A limited targeted LC–MS/MS panel was used to evaluate 12 selected non-anthocyanin phenolic analytes, including procyanidin dimers, a flavan-3-ol, flavonols, and phenolic acids; the contribution of the pH-sensitive anthocyanin fraction was not resolved. Among the tested metal ions, Fe^3+^ produced the strongest chromogenic response. The optimized L-cysteine pretreatment–Fe^3+^ mordanting–polyphenol dyeing sequence produced a ΔE* of 55.42. Adsorption experiments empirically described the uptake behavior of the polyphenol extract, whereas complementary surface, spectroscopic, elemental, diffraction, thermal, and wettability analyses were consistent with the presence of a relatively uniform Fe-containing polyphenol coating at the hair fiber interface. Neither the adsorption-model fits nor the individual characterization techniques uniquely resolved the underlying molecular mechanism. Preliminary HET-CAM, single-exposure dermal, and acute eye irritation assessments showed no obvious acute irritation under the tested conditions. An exploratory image-based analysis examined whether standardized hair tress photographs could approximate a predefined colorimetric score. Because only 30 independent original samples were available and no external validation was performed, this analysis was treated solely as a proof of concept. This laboratory proof-of-concept extends established Fe^3+^–polyphenol assembly chemistry to a compositionally complex *A. melanocarpa* extract for hair fiber coloration. Further work is required to clarify anthocyanin behavior, simplify the sequential protocol, and benchmark its performance against representative commercial hair dyes.

## 1. Introduction

With the continued growth of the personal-care and cosmetic industries, hair dyeing has become a common practice for managing appearance and masking visible signs of hair aging. At present, mainstream commercial hair dyes are still dominated by oxidative synthetic formulations containing aniline derivatives such as p-phenylenediamine (PPD), ammonia, and hydrogen peroxide [1,2]. Frequent exposure to these products may damage keratin disulfide bonds, cause lipid loss, impair the mechanical properties of hair fibers, and induce contact dermatitis, systemic allergic reactions, or other potential health risks [3,4]. In addition, wastewater generated during the production and rinsing of synthetic dyes may contain persistent organic compounds and increase the environmental burden. Therefore, developing plant-derived dyeing systems with improved biocompatibility, environmental compatibility, and color stability has become an important research direction in green chemistry, materials science, and bio-based product development [5,6,7].

Plant phenolic compounds have been investigated as natural colorants and interfacial coating components because many contain catechol, galloyl, or related oxygen-donor motifs that can interact with keratin and coordinate metal ions [8,9,10]. Fe^3+^–polyphenol coordination and metal–phenolic network assembly are well-established materials concepts rather than a new chemistry introduced in the present study. In the context of hair dyeing, Geng et al. reported interfacial metal–phenolic assembly using a defined phenolic building block and different metal ions, establishing the feasibility of tunable MPN-based coloration and washing-resistant deposition on hair fibers [11]. Subsequent work by Fang et al. introduced a thiol-assisted strategy to enhance interfacial and internal deposition of metal–polyphenol networks [12]. These studies demonstrate that both Fe^3+^–polyphenol hair coloration and engineered MPN deposition have been investigated previously.

The present study addresses a different, feedstock-specific question. Previous systems used defined phenolic ligands and/or deliberately engineered deposition strategies, whereas an *Aronia melanocarpa* extract is a compositionally complex mixture containing pH-sensitive anthocyanins, procyanidins, flavan-3-ols, flavonols, phenolic acids, and other uncharacterized components. Such complexity introduces uncertainty in ligand identity, relative abundance, competing interactions, and batch-to-batch reproducibility. The contribution of this study therefore lies in evaluating whether this extract can serve as a source-specific multiligand feedstock and in integrating its recovery with hair dyeing process evaluation, rather than in proposing Fe^3+^–polyphenol assembly as a new hair dyeing concept. Because no matched comparison with previously reported Fe^3+^–polyphenol hair dye systems was performed, no claim of superiority over those systems is made.

*Aronia melanocarpa* contains a compositionally complex mixture of anthocyanins, procyanidins, flavan-3-ols, flavonols, and phenolic acids [13,14,15,16]. Procyanidins are important contributors to the pronounced astringency of the fruit and contain multiple catechol-type oxygen-donor sites that may participate in Fe^3+^ association. Anthocyanins are also abundant constituents of *A. melanocarpa*, but their chromophore structures, visible-light absorption, and metal-binding behavior are strongly dependent on pH. Consequently, pH-dependent structural interconversion, hydration, degradation, and metal association of anthocyanins may alter both color development and their contribution to the overall Fe^3+^–polyphenol assembly. The observed dyeing behavior of a crude berry extract should therefore not be attributed exclusively to procyanidins, to the selected non-anthocyanin compounds detected by targeted analysis, or to any single phenolic class. The analytical distinction between anthocyanin and non-anthocyanin fractions is particularly important in this context. A limited target panel of selected non-anthocyanin compounds cannot be treated as a complete representation of the extract, and the presence of calibration curves does not by itself establish the abundance or dominance of the corresponding analytes. Comprehensive interpretation therefore requires either direct quantitative measurement of both fractions or explicit recognition of the restricted analytical scope.

The use of a compositionally complex plant extract also differs from the use of a purified phenolic ligand. Variations in cultivar, harvest season, storage, extraction conditions, and production batch may alter both the abundance and reactivity of the available ligands. In addition, the relative contribution of pH-sensitive anthocyanins and more persistent non-anthocyanin phenolics may change during processing. Source-specific extraction, compositional characterization, and process optimization are therefore required before an *A. melanocarpa* extract can be evaluated as a multiligand feedstock for Fe^3+^-mediated hair fiber coating formation.

Practical translation presents an additional challenge. Many commercial oxidative hair dye products are supplied as two components that are mixed immediately before use but applied to the hair in a single application. By contrast, sequential pretreatment, mordanting, and dyeing allow the functions of cuticle conditioning, Fe^3+^ anchoring, and phenolic deposition to be examined and optimized separately, but they also increase treatment time, rinsing requirements, and operational complexity. A sequential protocol may therefore be useful for laboratory-scale mechanistic evaluation and process screening, but it should not automatically be regarded as more convenient or commercially competitive than a single-application product. Direct comparison with representative commercial oxidative and plant-derived hair dyes under matched conditions is required before practical superiority can be assessed.

Because the composition of plant extracts may vary among raw-material and processing batches, reproducible evaluation of the resulting color remains important. Instrumental colorimetry was the primary quantitative method used in this study and was sufficient for evaluating dyeing performance. The image-based analysis was not required to establish the extraction, coating, or dyeing conclusions [17,18,19]. It was included only to explore whether standardized hair tress photographs could approximate a predefined colorimetric score and thereby provide a possible low-cost screening adjunct when instrument-based measurements are unavailable. Given the limited number of independent real samples, this component was treated solely as a proof-of-concept analysis and not as a principal contribution or a deployable quality-control system.

**Figure 1:**
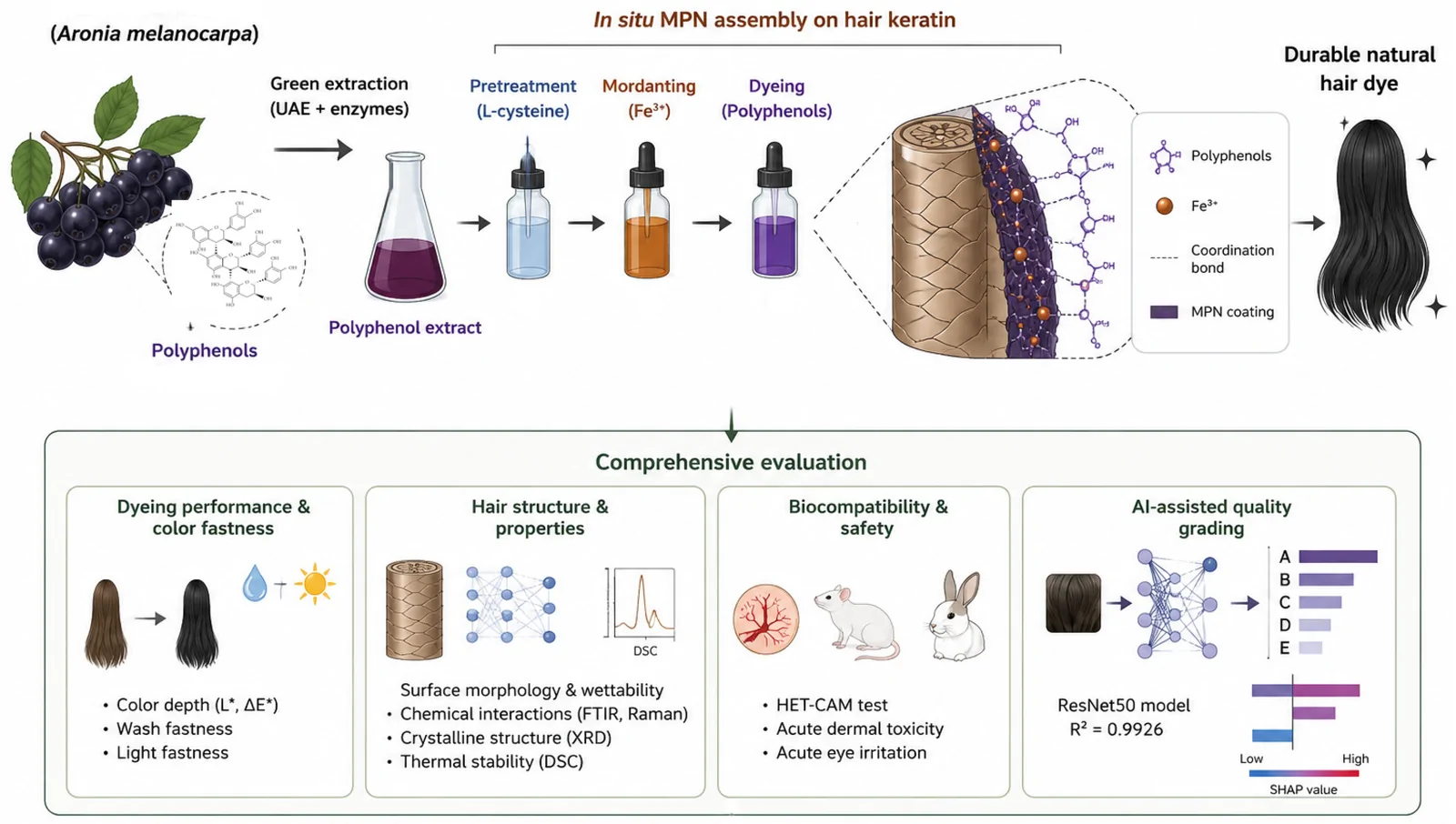
Study workflow for extending established Fe^3+^–polyphenol assembly chemistry to an *Aronia melanocarpa* extract for hair fiber coloration. The workflow integrates ultrasound-assisted recovery of phenolic constituents, sequential L-cysteine pretreatment, Fe^3+^ mordanting using ammonium ferric citrate, and polyphenol dyeing, followed by application-oriented evaluation of color performance, environmental stability, hair fiber properties, preliminary acute-irritation screening, and exploratory image-based analysis. The schematic represents a working process model for this study and does not imply that the exact molecular coordination structure of the Fe^3+^–polyphenol assembly has been fully resolved.

Against this established background, the objective of this study was not to introduce Fe^3+^–polyphenol assembly as a new materials concept, but to evaluate an *A. melanocarpa*-specific resource-conversion application. We optimized ultrasound-assisted cellulase–pectinase extraction, performed targeted phenolic profiling, and examined whether the recovered extract could function as a multiligand source in a sequential L-cysteine pretreatment–Fe^3+^ mordanting–polyphenol dyeing process (Figure 1). Color performance and hair mechanical properties were considered jointly during process optimization. Complementary surface, spectroscopic, elemental, diffraction, and thermal analyses were then used to assess whether the observations were consistent with the formation of an Fe-containing polyphenol coating at the hair fiber interface. Preliminary acute-irritation assays and a proof-of-concept image-based quality-evaluation component were included as application-oriented assessments. Thus, the contribution of this study lies in linking phenolic recovery and non-food valorization of *A. melanocarpa* with a hair fiber coating application, rather than in claiming novelty for the general Fe^3+^–polyphenol MPN concept. Throughout the Results and Discussion, outcomes directly supported by the figures and tables are described as experimental observations, whereas molecular explanations not directly measured in this study are presented only as possible interpretations or working hypotheses.

## 2. Results and Discussion

### 2.1. Optimization of Phenolic Extraction and Limited Targeted Analysis of Selected Phenolics in Aronia melanocarpa Extract

*Aronia melanocarpa* fruits are rich in polyphenols, among which condensed tannins, particularly procyanidins B1 and B2, are major contributors to their pronounced astringency [20,21]. These polyphenols can interact with salivary proteins through hydrophobic association and hydrogen-bond-mediated crosslinking, leading to protein precipitation and the characteristic rough mouthfeel associated with astringency [22]. The same catechol and pyrogallol motifs that underlie astringency also provide strong metal-coordinating ability, enabling chelation with transition-metal ions such as Fe^3+^ and subsequent formation of metal–phenolic networks (MPNs) [23,24]. Therefore, efficient enrichment of these astringency-related polyphenols is a prerequisite for converting a sensory limitation into a functional material advantage.

In this study, ultrasound-assisted combined enzymatic extraction was used to recover polyphenols from *A. melanocarpa*. Plant cell walls, composed mainly of cellulose frameworks and pectin-rich matrices, represent a key barrier that restricts the release of intracellular and cell-wall-bound polyphenols. Cellulase and pectinase can degrade these components in a complementary manner, thereby facilitating the liberation of phenolic compounds [25,26]. Single-factor experiments (Figure S1a,b) showed that the optimal cellulase-to-pectinase ratio was 5:5 and that the optimal total enzyme dosage was 3%. When the enzyme ratio deviated from 5:5, insufficient degradation of the composite cell-wall structure may have limited polyphenol release. When the enzyme dosage exceeded 3%, the extraction yield reached a plateau, which may be related to substrate saturation and competitive adsorption of polyphenols by enzyme proteins [27,28]. The highest polyphenol yield was obtained at a solid–liquid ratio of 1:50 (Figure S1c). Although a further increase in solvent volume could improve mass transfer to some extent, it may also reduce process efficiency during subsequent concentration. Therefore, 1:50 was selected as a practical balance between extraction efficiency and process economics.

**Figure 2:**
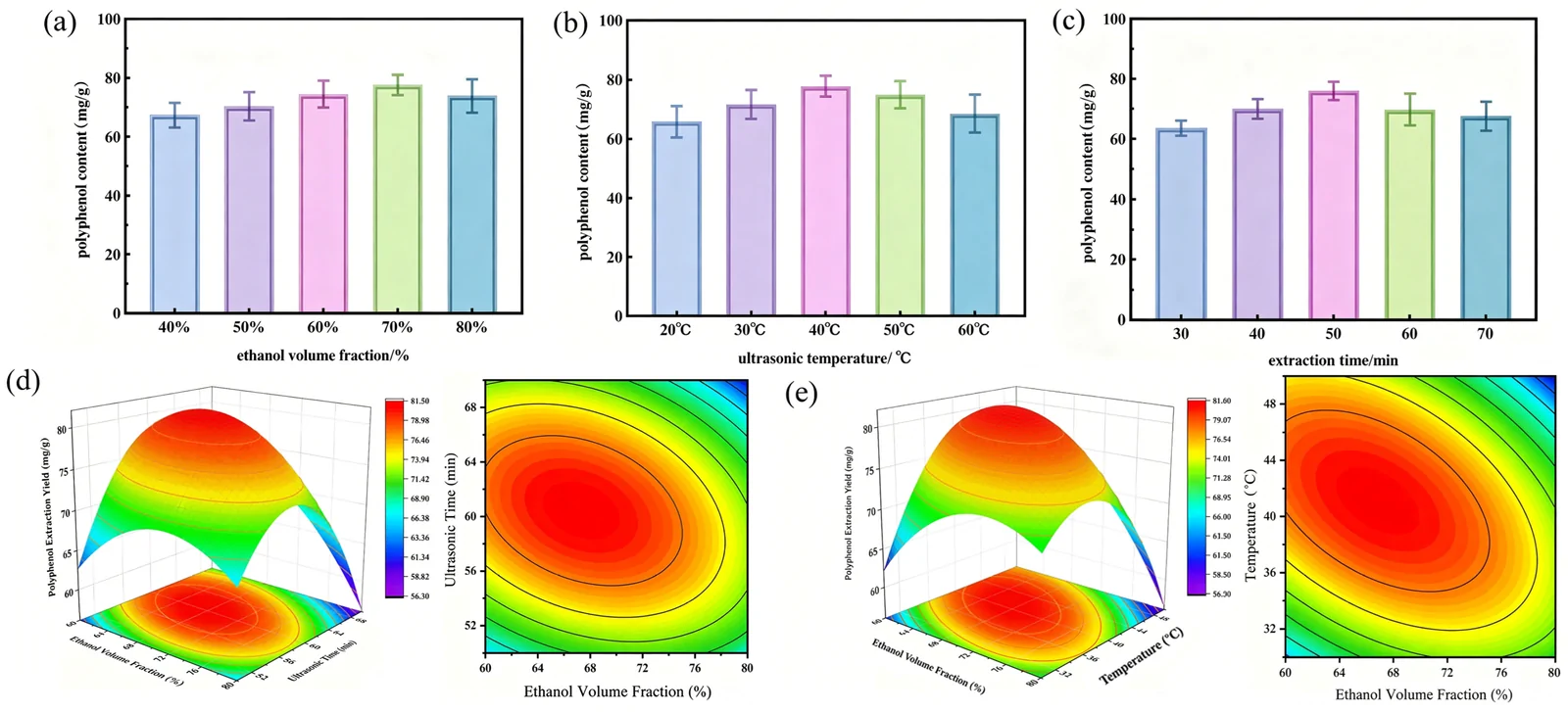
Optimization of ultrasound-assisted enzymatic extraction of polyphenols from *Aronia melanocarpa*. (**a**–**c**) Effects of ethanol concentration, ultrasonication temperature, and ultrasonication time on polyphenol yield. (**d**,**e**) Response surface and contour plots showing the interaction effects of ethanol concentration with extraction temperature (AC) and extraction time (AD), respectively. Data in (**a**–**c**) are presented as mean ± SD (*n* = 3). Error bars represent standard deviations. Statistical significance was analyzed using Duncan’s multiple range test at *p* < 0.05.

Solvent polarity played a critical role in polyphenol recovery. As shown in Figure 2a, the polyphenol yields first increased and then decreased with increasing ethanol volume fraction, with an optimum at 70%. A lower ethanol concentration may be insufficient for dissolving less-polar oligomeric procyanidins, whereas excessive ethanol may reduce the solubility of some flavonoid glycosides and phenolic acids [29]. Ultrasonication temperature (Figure 2b) and Ultrasonication time (Figure 2c) also showed single-peak response patterns, with optimal values of 40 °C and 60 min, respectively. Moderate heating can enhance molecular diffusion and cell-wall disruption, whereas higher temperatures (>50 °C) may accelerate polyphenol oxidation, degradation, or secondary polymerization of procyanidin-derived phenolics [30]. Similarly, prolonged ultrasonication beyond 60 min may promote sonochemical oxidation and structural damage of phenolic compounds. These results suggest that mild Ultrasonication conditions are preferable for balancing extraction efficiency with polyphenol stability.

Box–Behnken response surface analysis further revealed the nonlinear effects of extraction parameters on polyphenol yield (Tables S1 and S2). Analysis of variance showed that the overall regression model was highly significant (*p* < 0.0001), while the lack-of-fit term was not significant (*p* = 0.1168), indicating that the model adequately fitted the experimental data and could be used to predict polyphenol yield. Based on the F values from the ANOVA, the solid–liquid ratio and ethanol volume fraction were the dominant factors affecting polyphenol extraction, suggesting that solvent amount and solvent polarity played major roles in mass transfer and phenolic solubilization, while ultrasonic temperature and extraction time mainly exerted secondary regulatory effects.

Among the interaction terms, the interactions between ethanol volume fraction and ultrasonic temperature (AC) and between ethanol volume fraction and ultrasonic time (AD) were highly significant (*p* < 0.0001), as shown in Figure 2d,e. The corresponding contour plots showed elliptical patterns, indicating pronounced coupling effects between these variables. At relatively high ethanol concentrations, moderate heating may enhance the dissolution of less-polar procyanidin oligomers; however, the combination of high temperature and prolonged extraction time may accelerate polyphenol oxidation and consequently reduce extraction yield. In comparison, the interactions involving the solid–liquid ratio were generally weaker than AC and AD, suggesting that the solid–liquid ratio mainly influenced the extraction process through an independent mass-transfer effect. This feature may be useful for process scale-up, where solvent loading can be adjusted as a relatively independent parameter.

After model optimization and experimental validation, the final extraction conditions were determined as follows: cellulase-to-pectinase ratio of 5:5, total enzyme dosage of 3%, ethanol volume fraction of 70%, solid–liquid ratio of 1:50 g/mL, ultrasonication temperature of 40 °C, and ultrasonic time of 60 min. Under these conditions, the extracted total phenolic yield reached 82.315 ± 0.42 mg GAE/g dry fruit, which was close to the model-predicted value of 81.068 mg GAE/g dry fruit, with a relative deviation of less than 1.6%. Both values were calculated relative to the initial mass of dried fruit powder used for extraction, rather than the mass of the recovered extract. This agreement supported the predictive adequacy of the response surface model under the tested conditions. Compared with previously reported single-solvent extraction methods, which generally yielded approximately 40–55 mg/g, and single-enzyme-assisted extraction methods, which yielded approximately 60–65 mg/g [25,31], the present combined enzymatic–ultrasound strategy increased the yield by approximately 25–35%, demonstrating improved recovery of total phenolics from *A. melanocarpa* under the tested conditions.

A targeted LC–MS panel was used to screen 12 preselected non-anthocyanin phenolic standards (Table 1). The panel was selected on the basis of reported occurrence in *A. melanocarpa*, analytical-standard availability, and the presence of oxygen-donor motifs potentially relevant to Fe^3+^ binding. The calibration curves describe the responses of the analytical standards; they do not establish the relative abundance or quantitative dominance of these compounds in the crude extract. Anthocyanins were not included in the present panel and were therefore not assessed. The detected compounds were grouped into four structural categories: procyanidin dimers, including procyanidin B1 and procyanidin B2; a monomeric flavan-3-ol, epicatechin; flavonols and their glycosides, including quercetin, kaempferol, rutin, and hyperoside; and phenolic acids, including chlorogenic acid, caffeic acid, protocatechuic acid, ferulic acid, and vanillic acid. This profile is broadly consistent with reported phenolic constituents of *A. melanocarpa* [32].

From the perspective of coordination chemistry, the detected phenolic profile provides a plausible molecular basis for subsequent MPN assembly. Procyanidin B1 and B2 are B-type flavan-3-ol dimers containing multiple phenolic hydroxyl groups, including catechol-bearing B rings, which can participate in Fe^3+^ coordination [33]. Epicatechin is a monomeric flavan-3-ol and represents an additional catechol-containing ligand. Quercetin contains several potential metal-binding sites, including the 3-hydroxyl/4-carbonyl site and the catechol moiety on the B ring, whereas chlorogenic and caffeic acids also contain catechol-type oxygen-donor motifs [34,35]. The detected constituents provide several plausible oxygen-donor motifs for Fe^3+^ binding. Procyanidins B1/B2, epicatechin, caffeic acid, and chlorogenic acid contain catechol-type O,O donor sites, whereas quercetin may coordinate through the B-ring catechol and/or the 3-hydroxyl/4-carbonyl site. Because the mordant was ammonium ferric citrate and the dye was a crude extract, Fe–citrate, Fe–phenolate, and mixed-ligand species may coexist. The present data therefore support a multiligand interfacial association model but do not identify the exact stoichiometry or molecular structure of the complexes.

Quercetin, chlorogenic acid, and several other phenolic compounds included in the target panel have been reported to possess radical-scavenging activity [35]. However, the present analysis did not provide validated sample-specific concentrations for these analytes, and the antioxidant capacity of the complete extract or treated hair fibers was not measured. Their literature-reported antioxidant properties therefore provide compositional context only and are not used here as direct evidence that the formulation protected hair through radical scavenging.

### 2.2. Construction and Process Optimization of the Polyphenol–Fe^3+^ Natural Dyeing System

For a natural polyphenol-based dyeing system, efficient color development must be balanced with the preservation of hair fiber integrity. Strong pretreatment can facilitate cuticle opening and polyphenol penetration, but excessive chemical treatment may irreversibly disrupt the keratin disulfide network and lead to fiber embrittlement. Therefore, in this study, both color depth and hair fiber mechanical properties were used as evaluation criteria for screening the mordant ion, optimizing the dyeing sequence, and refining the three-step dyeing process.

**Figure 3:**
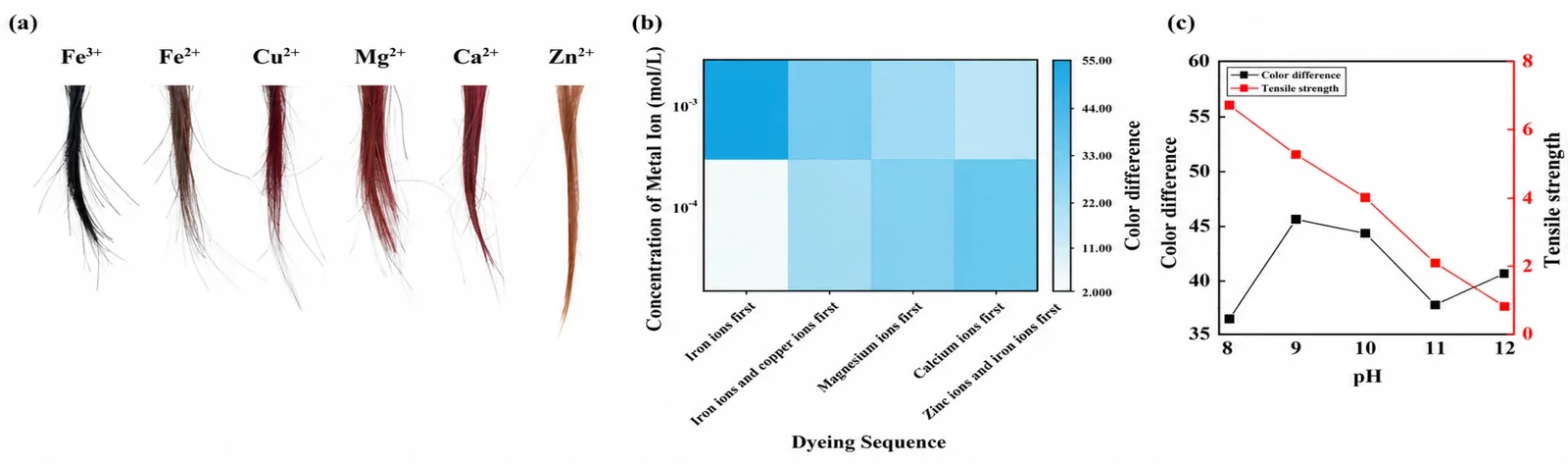
Optimization of the sequential L-cysteine pretreatment–Fe^3+^ mordanting–polyphenol dyeing process. (**a**) Representative images of bleached hair tresses dyed with *A. melanocarpa* polyphenols using different metal ions as mordants, including Fe^3+^ supplied by ammonium ferric citrate. (**b**) Comparison of L* and ΔE* values among different dyeing sequences: direct dyeing, simultaneous mordanting and dyeing, dyeing followed by mordanting, and mordanting followed by dyeing. (**c**) Effect of pretreatment pH on color development and tensile strength.

The electronic structure of metal ions directly affects the visible-light absorption and macroscopic color of polyphenol–metal complexes. Six metal ions, including Fe^3+^, Fe^2+^, Cu^2+^, Mg^2+^, Ca^2+^, and Zn^2+^, were compared as mordants (Figure 3a, Table 2). Within the standardized bleached-hair model, the blue-black shade was selected as the technical target because it combined the lowest L*, a large ΔE*, visual uniformity, and acceptable mechanical performance. This selection was not based on consumer-preference testing, and no inference is made regarding market acceptance or the optimal shade for different starting hair colors. This performance resulted in the largest color difference among the tested metal ions, with ΔE* = 53.08 for Fe^3+^ compared with 35.76 for Fe^2+^, 41.91 for Cu^2+^, 29.41 for Mg^2+^, 10.53 for Ca^2+^, and 9.99 for Zn^2+^ under the same experimental conditions.

Experimentally, Fe^3+^ produced the lowest L* value and the largest ΔE* among the tested metal ions, resulting in the darkest blue-black shade under the present conditions. One possible interpretation is that Fe^3+^ forms strongly absorbing charge-transfer complexes with oxygen-donor phenolic ligands [23,24,36]. However, the present study did not directly measure the UV–visible absorption spectra, metal speciation, ligand-to-metal stoichiometry, hydrolysis state, or aggregation behavior of the different metal–phenolic systems. Differences in ligand affinity, ligand exchange, metal hydrolysis, complex solubility, and particle formation may also have contributed to the observed colors. Fe^3+^ was therefore selected on the basis of its empirical chromogenic performance rather than on a uniquely demonstrated electronic mechanism. Among Fe^3+^-containing reagents, ammonium ferric citrate was selected as the Fe^3+^ source for subsequent mordanting experiments. This selection was based on its aqueous compatibility, practical handling characteristics, and suitability for a mild hair-treatment process. Citrate-associated Fe^3+^ species may influence Fe^3+^ availability and aqueous speciation during mordanting, which may contribute to controlling the interaction environment between Fe species and phenolic components. However, the present study did not perform a systematic comparison among different Fe^3+^ salts, such as FeCl_3_, Fe(NO_3_)_3_, or Fe_2_(SO_4_)_3_. Therefore, ammonium ferric citrate is considered the selected Fe^3+^ reagent for this experimental system rather than a universally superior Fe^3+^ source.

After Fe^3+^ was selected as the mordant, the dyeing sequence became a key factor affecting dye uptake and color uniformity. As shown in Figure 3b and Table S3, mordanting followed by dyeing produced greater color depth (L* = 26.31, ΔE* = 55.42) and better visual uniformity than direct dyeing, simultaneous mordanting and dyeing, or dyeing followed by mordanting.

The sequence dependence may reflect differences in where and when Fe^3+^ species and phenolic components associate. During simultaneous mordanting and dyeing, rapid Fe^3+^–phenolic association in the bulk solution may generate solution-phase assemblies before the components reach the hair surface, potentially reducing the fraction available for uniform interfacial deposition [23,24,37]. During dyeing followed by mordanting, preadsorbed phenolic components may interact with subsequently added Fe^3+^ both at the hair surface and in the surrounding solution, resulting in concurrent interfacial and bulk-phase association [11,23,24].

By contrast, mordanting followed by dyeing may increase the local availability of hair-associated Fe species before application of the polyphenol extract, thereby favoring subsequent interfacial deposition [11,38]. This interpretation is consistent with the greater color depth observed for this sequence. However, it should be regarded as a proposed working model: the present data do not establish the exact Fe^3+^ binding sites on keratin, the molecular coordination structure of the deposited species, or their penetration depth within the hair fiber.

After identifying mordanting followed by dyeing as the preferred sequence, key parameters of the three-step dyeing process were further optimized for L-cysteine pretreatment, Fe^3+^ mordanting, and polyphenol dyeing (Figure 3c; complete single-factor optimization results are shown in Figure S2). The main role of the pretreatment step was to moderately modify the hair surface structure, thereby improving subsequent Fe^3+^ anchoring and polyphenol penetration. L-cysteine, as a thiol-containing reducing agent, can partially reduce surface disulfide bonds in keratin under mildly alkaline conditions, loosening the cuticle structure and increasing the accessibility of active sites and diffusion channels [11,39]. As pretreatment pH increased, the color difference value (ΔE*) gradually increased, indicating that alkaline activation facilitated dyeing. However, when the pH exceeded 10, the tensile strength decreased markedly compared with the optimal condition, indicating a trade-off between enhanced color development and fiber mechanical preservation under stronger alkaline treatment. This mechanical deterioration may be associated with accelerated β-elimination of disulfide bonds under strongly alkaline conditions, generating irreversible degradation products such as dehydroalanine [36]. Therefore, considering both color development and mechanical integrity, the optimal pretreatment conditions were determined to be pH 9, 8% L-cysteine, 45 °C, and 40 min.

During the Fe^3+^ mordanting step, an ammonium concentration of 8% produced the most favorable response in the concentration series, while 45 °C produced ΔE* = 52.37 and L* = 30.23 in the temperature series, while a mordanting temperature of 45 °C produced ΔE* = 52.37 and L* = 30.23 in the temperature series. Further increases in ammonium ferric citrate concentration were associated with reduced color development. This trend may reflect changes in Fe^3+^ speciation, hydrolysis, or solution-phase association [36,40], which may in turn have affected the amount and distribution of Fe species available for interfacial deposition [11,38]. However, Fe(OH)_3_ formation, turbidity, particle size, and soluble-iron speciation were not directly measured. A pH of 4.5 produced the most favorable color response in the tested pH series. A balance between Fe^3+^ coordination availability and hydrolysis is a possible interpretation [36,40], but the underlying solution equilibria were not independently determined. Accordingly, the optimal mordanting conditions were determined as pH 4.5, 8% ammonium ferric citrate, 45 °C, and 40 min.

During the polyphenol dyeing step, a polyphenol concentration of 20% achieved both high color depth (L* = 21.84, ΔE* = 59.69) and relatively good mechanical stability of the hair fibers. Lower concentrations may have limited the availability of phenolic components, whereas the reduced color development at the higher concentration may reflect solution-phase association, viscosity-related mass-transfer limitations, saturation of accessible hair-associated regions, or other formulation effects. These possibilities were not experimentally distinguished. A pH of 5.5 produced the most favorable overall response in the tested pH series. A pH-dependent balance between phenolic speciation and Fe^3+^ association is a possible interpretation [36,40]; however, the protonation states of individual phenolic constituents, anthocyanin speciation, and coordination with hair-associated Fe species were not directly measured. Therefore, the optimal dyeing conditions were determined as pH 5.5, 20% polyphenol solution, 45 °C, and 40 min.

Taken together, the optimized dyeing process consisted of three sequential steps: L-cysteine pretreatment, Fe^3+^ mordanting, and polyphenol dyeing. This process produced deep coloration (ΔE* = 55.42, L* = 26.31) while maintaining hair fiber mechanical performance. The three pH values corresponded to separate treatment baths, and the hair tresses were rinsed between successive steps. The polyphenol extract was introduced only in the final dyeing bath at pH 5.5 and was therefore not directly exposed to the alkaline pretreatment bath. Nevertheless, residual local pH, pre-anchored Fe^3+^ species, and the pH-dependent equilibria of anthocyanins may influence the final color. Because anthocyanins were not quantified in the present targeted analysis, their specific contribution remains unresolved. Mild alkaline pretreatment activated the hair surface, weakly acidic mordanting maintained Fe^3+^ availability for coordination, and near-neutral dyeing promoted polyphenol assembly at the hair interface. Under the experimental conditions, separating pretreatment, mordanting, and dyeing enabled independent control of hair-surface activation, Fe^3+^ anchoring, and polyphenol deposition. However, the three 40 min treatments and intermediate rinsing steps increase application time and operational complexity compared with many commercial single-application products. The present sequence should therefore be regarded as a mechanistic and formulation prototype; future work should evaluate process consolidation without compromising color depth or fiber integrity.

### 2.3. Empirical Uptake Behavior of Aronia melanocarpa Polyphenols by Hair Fibers and Environmental Stability of the Sequential Dyeing System

After optimization of the dyeing procedure, kinetic and equilibrium experiments were used to describe the time- and concentration-dependent uptake of the *Aronia melanocarpa* polyphenol extract by hair fibers. The model fits were treated as empirical descriptions of the observed data rather than as direct evidence of a unique adsorption mechanism. The kinetic and isotherm experiments evaluated the interaction between the polyphenol solution and hair fibers, whereas the washing and light-exposure experiments described later in this section were performed on hair treated using the complete sequential dyeing protocol. Accordingly, the adsorption-model fits were not used to infer Fe^3+^-bridged adsorption or metal–phenolic network formation.

**Figure 4:**
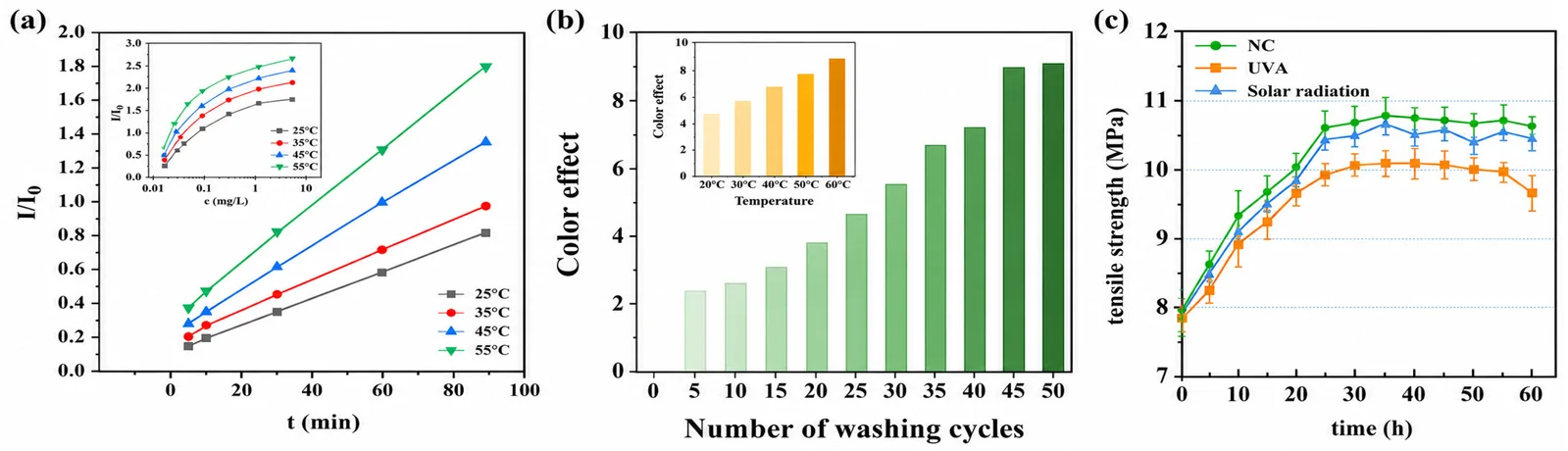
Empirical uptake behavior of *Aronia melanocarpa* polyphenols by hair fibers and environmental stability of sequentially dyed hair. (**a**) Linearized pseudo-second-order representation of polyphenol uptake by hair fibers at different temperatures, with the inset showing the linearized Langmuir representation at 45 °C. These model plots are used to describe the experimental data and are not interpreted as proof of a unique adsorption mechanism. (**b**) Changes in ΔE* of sequentially dyed hair fibers during repeated washing at 45 °C and 55 °C. (**c**) Changes in color difference and tensile strength of dyed and bleached hair fibers under UVA irradiation and simulated sunlight exposure.

The kinetic curves showed a two-stage uptake pattern, consisting of a relatively rapid increase during the first 0–50 min followed by a gradual approach to an apparent plateau after approximately 100 min (Figure 4a and Table S4). The linearized pseudo-second-order representation produced R^2^ values of 0.9641–0.9915 over the tested temperature range. These values indicate that the equation described the time-dependent data reasonably well within the present linearized representation. However, because the current assessment relied primarily on a linearized form and its R^2^ value, the fitted parameters and apparent model performance should be interpreted cautiously [41]. Agreement with the pseudo-second-order equation does not distinguish physical adsorption from chemical adsorption, identify a unique rate-limiting step, or establish the presence of a specific type of molecular bond [42,43].

The observed uptake may reflect a combination of external mass transfer, fiber-associated diffusion, and interfacial association. Their relative contributions were not independently resolved in the present study, and the pseudo-second-order fit cannot identify which of these processes controlled the overall uptake [43]. Therefore, the kinetic fit should not be interpreted as evidence that chemical interactions dominated over diffusion or other transport processes.

The model-derived equilibrium uptake parameter Q_e,2_ increased from 0.8434 mg/g at 25 °C to 1.7687 mg/g at 55 °C. This trend indicates greater apparent polyphenol uptake at higher temperatures under the tested conditions. However, the observed temperature dependence may reflect a combination of changes in molecular diffusion, solution properties, fiber swelling, and the accessibility of hair-associated regions. The available kinetic data do not distinguish among these possible contributions and do not establish a specific adsorption mechanism.

The equilibrium data were also represented using the Langmuir and Freundlich equations (Figure S3 and Table S5). Across the tested temperatures, the linearized Langmuir representation produced R^2^ values of 0.984–0.994, whereas the Freundlich representation produced R^2^ values of 0.937–0.976. Thus, within the investigated concentration and temperature ranges, the selected Langmuir representation provided a closer empirical description of the equilibrium data. This comparison does not demonstrate that the hair fibers contain completely homogeneous adsorption sites, that actual molecular monolayer coverage occurred, or that particular keratin functional groups acted as defined coordination nodes.

The fitted Qm and KL values should therefore be regarded as model-dependent parameters rather than as direct measurements of actual monolayer coverage or molecular binding energy. Because the current comparison was based primarily on linearized fitting coefficients, without nonlinear regression, additional error statistics, or residual analysis, both the apparent model preference and the fitted parameter values should be interpreted cautiously [44].

Taken together, the kinetic and equilibrium models summarize the uptake behavior of the *A. melanocarpa* polyphenol extract under the tested conditions, but they do not establish chemisorption or any other unique molecular mechanism. Hydrogen bonding, hydrophobic association, and other possible phenolic–keratin interactions should therefore be regarded as working hypotheses that require evaluation using independent structural and interfacial evidence [9,10].

Repeated washing was used to evaluate the practical color retention of the dyed hair fibers. After 15 washing cycles at 45 °C, the cumulative color difference in the dyed hair fibers was approximately ∆E* = 2.39, below the commonly used color-difference perceptibility threshold of ∆E* ≥ 3.0 [45] (Figure 4b). This result indicates that the deposited Fe-containing polyphenol layer maintained relatively stable coloration under the tested washing conditions. When washing was extended to 20 cycles, ΔE* increased to 3.75, indicating progressive color loss during repeated washing. At 55 °C, washing accelerated color loss, and ∆E* reached 3.37 after 15 cycles, exceeding the perceptible threshold. This temperature-dependent fading behavior indicates that high-temperature washing may disrupt reversible interfacial components and reduce color stability [46]. Therefore, washing at temperatures not exceeding 45 °C may help maintain the color durability of the polyphenol-based dyeing system.

Light-stability tests further showed that the dyed hair fibers maintained relatively stable color under both UVA irradiation and simulated sunlight exposure. During the first 50 h of exposure, the ΔE* values remained below 3.0, whereas after 55 h they exceeded the perceptible threshold (Figure 4c). The observed light stability may have several possible explanations. Fe^3+^–phenolic association and the deposited Fe-containing polyphenol layer may influence the photochemical behavior of the chromogenic components and their exposure to incident light [47,48]. However, coating UV transmittance, photoproduct formation, ROS generation, and radical-scavenging activity under the applied exposure conditions were not measured. These mechanisms should therefore be regarded as hypotheses rather than as experimentally established causes of the observed color retention.

Mechanical testing after light exposure showed that, after 60 h of irradiation, the tensile strength of the polyphenol–Fe^3+^-treated hair fibers remained approximately 10–11 MPa, which was higher than that of the bleached undyed control under the same exposure conditions (Figure 4c). The decrease in elongation at break was also less pronounced in the treated group. Photo-oxidative changes in keratin represent one possible contributor to the mechanical deterioration of bleached hair [48]. However, the present experiments did not directly determine whether the deposited layer acted as a physical barrier or whether residual phenolic constituents reduced oxidative stress. The results therefore show an association between the treatment and retention of mechanical properties under the tested light-exposure conditions, but they do not establish the underlying protective mechanism.

### 2.4. Multimodal Evidence for Interfacial Fe^3+^–Polyphenol Association and Hair Fiber Changes

The targeted LC–MS analysis described in Section 2.1 included 12 selected phenolic constituents, comprising procyanidin B1, procyanidin B2, epicatechin, quercetin, rutin, hyperoside, kaempferol, chlorogenic acid, caffeic acid, protocatechuic acid, ferulic acid, and vanillic acid. Within this targeted set, procyanidin B1, procyanidin B2, and epicatechin contain catechol-type B-ring structures; quercetin contains both a catechol moiety and a potential 3-hydroxy-4-keto metal-binding site; and caffeic acid, chlorogenic acid, and protocatechuic acid contain additional catechol-type oxygen-donor motifs. Rutin and hyperoside may also participate through the oxygen-donor groups retained in their flavonol structures. These compounds therefore represent plausible candidate ligands for Fe^3+^ association. However, because the present targeted analysis did not determine their relative contributions to Fe^3+^ binding and did not include anthocyanins, no individual compound can be identified as the dominant chelating component of the extract.

The most plausible coordination mode involves interactions between Fe^3+^ and oxygen-donor atoms in catechol-type or hydroxyl–carbonyl motifs. Fe^3+^ binding may promote deprotonation of phenolic oxygen atoms and generate O,O-coordination sites, allowing one Fe^3+^ center to associate with more than one phenolic ligand. Because ammonium ferric citrate was used as the Fe^3+^ source, ligand exchange between citrate-associated Fe^3+^ species and phenolic oxygen donors may also occur during mordanting and subsequent dyeing. In addition, hydrogen bonding, hydrophobic interactions, and electrostatic interactions may contribute to the association of the phenolic components with the keratin surface. The resulting material is therefore more appropriately regarded as a heterogeneous, multiligand, and potentially non-stoichiometric Fe^3+^–polyphenol interfacial assembly rather than a single, structurally defined molecular complex. The present experiments did not resolve the exact Fe^3+^ coordination number, ligand-to-metal stoichiometry, or three-dimensional coordination geometry.

To evaluate whether the observed surface and structural changes were consistent with interfacial Fe^3+^–polyphenol association, SEM, water contact angle analysis, ATR–FTIR, Raman spectroscopy, EDS, XRD, and DSC were combined as complementary characterization approaches. These techniques provide information at different length scales, including surface morphology, wettability, functional group environments, elemental distribution, apparent structural order, and thermal transition behavior. Nevertheless, these techniques provide complementary but non-unique evidence and do not, by themselves, resolve the exact coordination structure of the resulting Fe^3+^–polyphenol complexes.

**Figure 5:**
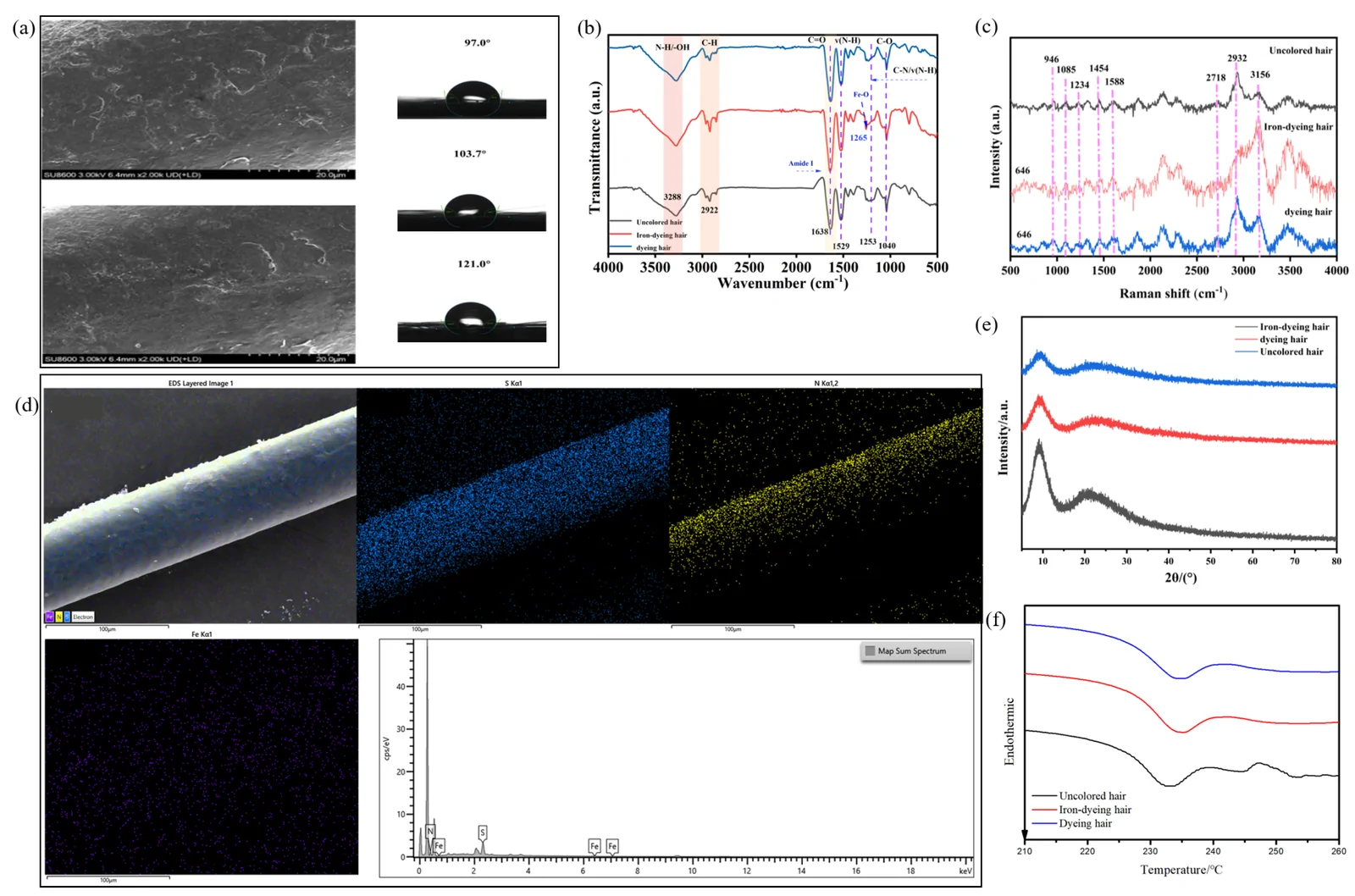
Multimodal characterization of interfacial Fe^3+^−polyphenol association and treatment-associated changes in hair fibers. (**a**) SEM images comparing the surface morphology of bleached undyed hair and polyphenol–Fe^3+^-treated hair, together with representative water contact angle images and values for bleached undyed hair, polyphenol-treated hair, and polyphenol–Fe^3+^-treated hair. (**b**) ATR–FTIR spectra showing treatment-associated changes in hydroxyl-, amide-, and oxygen-containing functional group regions. (**c**) Raman spectra showing vibrational changes after polyphenol and polyphenol–Fe^3+^ treatments, including a tentative Fe–O-related feature near 646 cm^−1^. (**d**) SEM image, EDS elemental maps, and EDS spectrum showing the distribution of C, O, and Fe in the analyzed surface region of polyphenol–Fe^3+^-treated hair. (**e**) XRD patterns of bleached undyed hair, polyphenol-treated hair, and polyphenol–Fe^3+^-treated hair, showing treatment-associated changes in the keratin-related diffraction profile and apparent relative crystallinity. (**f**) DSC thermograms of the three whole-hair samples, showing modest treatment-associated differences in keratin-associated thermal transition behavior; these differences are not interpreted as direct evidence of increased keratin ordering or structural restoration.

As shown in Figure 5a, SEM images of bleached undyed hair showed an irregular surface with lifted or damaged cuticle edges. After treatment with the polyphenol–Fe^3+^ system using the mordanting-followed-by-dyeing sequence, the hair surface appeared more continuously covered, and the cuticle boundaries appeared less irregular. These morphological observations are consistent with the deposition of an interfacial coating on the hair surface. However, SEM alone cannot determine whether the deposited material is chemically coordinated, nor can it establish penetration of the polyphenol–Fe^3+^ species into the cortical region.

The representative water contact angle measurements shown in Figure 5a further indicated treatment-dependent changes in surface wettability. The contact angle was 103.7° for bleached undyed hair, decreased to 97.0° after treatment with the polyphenol extract alone, and increased to 121.0° after polyphenol–Fe^3+^ treatment. The lower contact angle of the polyphenol-only group is consistent with the presence of exposed hydrophilic phenolic groups on the hair surface. The higher contact angle following Fe^3+^-mediated treatment suggests that coordination-associated aggregation or crosslinking altered the chemical composition and organization of the outer interfacial layer. This change may have reduced the accessibility of some hydrophilic groups and/or produced a more compact surface deposit. However, the contact angle data do not establish complete consumption of phenolic hydroxyl groups or a specific outward orientation of the aromatic structures.

ATR–FTIR spectra are presented in Figure 5b. Compared with bleached undyed and polyphenol-treated hair, the polyphenol–Fe^3+^-treated sample showed changes in the broad O–H/N–H stretching region at approximately 3200–3500 cm^−1^, together with changes in bands associated with oxygen-containing functional groups. These observations indicate that the local environment of hydroxyl- and oxygen-containing groups changed after Fe^3+^-mediated treatment. Changes in the C–O-rich and amide III regions, including the spectral feature near 1265 cm^−1^, may be associated with overlapping contributions from C–O, C–N, amide, and Fe–O-related vibrations. Therefore, this band should not be regarded as definitive proof of a specific Fe–O bond. Instead, the overall ATR–FTIR changes are interpreted as being consistent with altered hydrogen-bonding and metal–ligand interaction environments involving the polyphenol extract and the hair surface.

Raman spectroscopy provided complementary vibrational information, as shown in Figure 5c. Following polyphenol–Fe^3+^ treatment, a spectral feature near 646 cm^−1^ became more apparent than in the bleached undyed and polyphenol-only samples. This feature may be tentatively associated with Fe–O-related vibrations in iron–phenolic assemblies. Changes were also observed in the higher-wavenumber region associated with hydroxyl-containing structures. Because Raman bands in complex biological and plant-extract samples may overlap, the 646 cm^−1^ signal cannot independently establish a unique Fe–O coordination geometry. Its interpretation is strengthened only when considered together with the ATR–FTIR changes and the detection of surface-associated Fe by EDS.

The EDS results shown in Figure 5d confirmed the presence of Fe in the polyphenol–Fe^3+^-treated hair sample. Elemental mapping showed that Fe was distributed over the analyzed hair-surface region without obvious large, localized Fe-rich aggregates. The accompanying C and O maps were also broadly distributed across the same region. These observations support relatively uniform deposition of an Fe-containing polyphenol layer on the hair surface. However, EDS identifies elemental presence and spatial distribution only; it does not determine the oxidation state of iron, the molecular identity of the ligands, or the exact nature of the Fe–O coordination bonds.

XRD analysis was used to assess treatment-associated changes in the diffraction profiles of the whole hair samples, as shown in Figure 5e and Table S6. The calculated apparent relative crystallinity was 28.88 ± 0.32% for bleached undyed hair, 30.48 ± 0.34% for polyphenol-treated hair, and 37.36 ± 0.76% for polyphenol–Fe^3+^-treated hair. The polyphenol–Fe^3+^-treated sample therefore showed a numerically higher calculated apparent relative crystallinity than the other two groups. Relative to bleached undyed hair, this corresponded to an increase of 8.48 percentage points in calculated apparent relative crystallinity. These diffraction-profile changes are more appropriately described as changes consistent with increased calculated apparent relative crystallinity under the tested conditions, rather than as evidence of molecular reorganization of keratin.

Because the measurements were performed on whole treated hair fibers, the calculated values may reflect combined contributions from keratin-associated scattering, the deposited Fe-containing polyphenol layer, sample packing or orientation, baseline selection, and the peak-deconvolution procedure. The XRD-derived values should therefore be regarded as analysis-dependent descriptors of the diffraction profiles. They do not, by themselves, demonstrate restoration, reconstruction, or rearrangement of keratin domains.

DSC thermograms are shown in Figure 5f, and the corresponding thermal transition parameters are listed in Table S7. The denaturation peak temperature was 232.4 °C for bleached undyed hair, 234.1 °C for polyphenol-treated hair, and 234.3 °C for polyphenol–Fe^3+^-treated hair. The corresponding denaturation enthalpy values were 10.77, 10.49, and 10.83 J/g, respectively. Relative to bleached undyed hair, the polyphenol–Fe^3+^-treated sample therefore showed only a 1.9 °C increase in Td and a 0.06 J/g increase in ΔHd. Moreover, polyphenol-only treatment increased Td while decreasing ΔHd, indicating that the two parameters did not change in a simple, internally consistent pattern attributable to increased molecular ordering.

Because DSC was performed on whole treated hair samples, the mass-normalized ΔHd values may also be influenced by sample heterogeneity and the contribution of the deposited non-keratin layer. In addition, replicate variability was not reported for the DSC parameters. Accordingly, the DSC results are interpreted as showing modest treatment-associated changes in whole-sample thermal transition behavior. They do not independently demonstrate increased keratin ordering, molecular reorganization, or structural restoration.

Taken together, the SEM, wettability, ATR–FTIR, Raman, and EDS results support the presence of a relatively uniform Fe-containing polyphenol coating at the hair fiber interface. The XRD and DSC results indicate treatment-associated changes in diffraction-derived apparent relative crystallinity and whole-sample thermal transition behavior, respectively.

Collectively, these observations are consistent with interfacial Fe^3+^–polyphenol association involving multiple phenolic ligands and complementary noncovalent interactions with keratin. However, the present evidence does not uniquely establish the exact ligand composition, coordination stoichiometry, coordination geometry, or molecular architecture of the resulting complexes. Compound-specific binding experiments and coordination-sensitive analyses would be required to resolve these structural details.

### 2.5. Preliminary Acute-Irritation Screening of the Polyphenol–Fe^3+^ Dyeing System

Natural origin does not necessarily guarantee safety. Polyphenols may interact with biological macromolecules, and Fe^3+^ may participate in redox-active processes under some biological conditions. The safety-related experiments in the present study were therefore designed to characterize observable acute irritation endpoints under the specified test conditions. These experiments comprised HET-CAM acute vascular-irritation screening, a single 24 h occlusive dermal exposure followed by a 72 h observation period in mice, and an acute eye irritation assessment in rabbits. They were not designed to establish general biocompatibility or safety during repeated human use and did not evaluate cumulative irritation, skin sensitization, genotoxicity, chronic or systemic toxicity, dermal iron absorption, or responses in individuals with disorders of iron metabolism.

**Figure 6:**
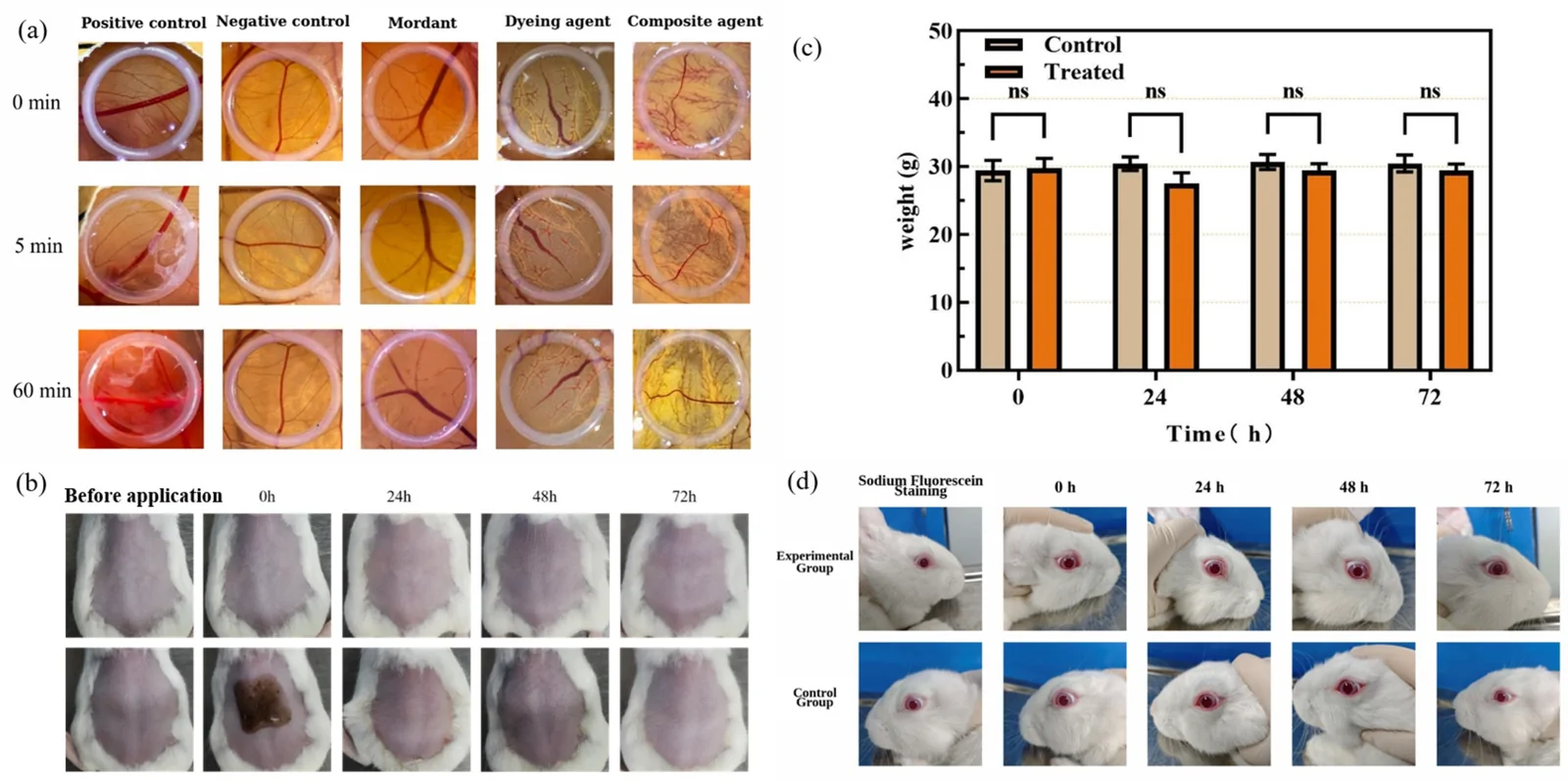
Preliminary acute-irritation screening of the polyphenol–Fe^3+^ hair dyeing system. (**a**) HET-CAM images showing acute vascular responses after treatment with saline as the negative control, SDS as the positive control, and the tested dyeing components at the specified observation times. (**b**) Representative dorsal-skin images of mice before and after a single 24 h occlusive dermal exposure. (**c**) Body-weight changes during the 72 h post-exposure observation period. “ns” indicates no statistically significant difference between the control and treated groups (*p* ≥ 0.05). (**d**) Fluorescein sodium staining images of rabbit eyes during the acute ocular observation period. These assays address acute endpoints only and do not establish repeated-use or long-term human safety.

The HET-CAM assay was used as an in vitro screening model because the vascular network of the chick chorioallantoic membrane is sensitive to chemical irritation and allows the observation of hemorrhage, coagulation, and vascular lysis [49]. In this study, 0.01% SDS was used as the positive control and 0.9% physiological saline as the negative control. Vascular responses were recorded at 0, 5, and 60 min after sample application (Figure 6a). The SDS-positive control induced typical vascular lysis and hemorrhage within 5 min. In contrast, vascular morphology remained intact throughout the 60 min exposure to the polyphenol dye solution and ammonium ferric citrate mordant, with no obvious hemorrhage, coagulation, or vascular contraction. The severity score was not significantly different from that of the saline negative control, and the samples were classified as non-irritant according to the Luepke scoring criterion [50].

Under the tested concentrations and exposure conditions, no observable hemorrhage, coagulation, or vascular lysis was detected after application of the polyphenol dye solution or ammonium ferric citrate mordant. These observations support the absence of observable acute vascular irritation within the applied HET-CAM model and scoring framework. However, this conclusion is limited to the acute vascular endpoints evaluated in this assay and does not address repeated exposure, skin sensitization, genotoxicity, systemic toxicity, or human scalp compatibility. The present results also do not identify a molecular mechanism responsible for the absence of visible membrane damage.

The acute dermal exposure test in mice was designed with reference to OECD 402. During the 24 h occlusive exposure and the subsequent 72 h observation period, no erythema, edema, eschar formation, or other visible skin irritation signs were observed on the dorsal skin of mice in either the test or control group (Figure 6b). The skin irritation scores remained within the non-irritant range of 0–0.4 according to the Draize scoring system [51]. Body weight monitoring showed only slight fluctuations within 24 h after exposure, with changes of less than 5%, similar to those observed in the control group after occlusive covering. Body weights in both groups returned to near-baseline levels at 48 and 72 h, and no significant intergroup differences were observed (Figure 6c). No mortality occurred during the experiment, and no signs of systemic toxicity, such as piloerection, hunched posture, abnormal gait, or behavioral abnormalities, were observed.

The mouse study involved a single 24 h occlusive dermal exposure followed by a 72 h observation period. The absence of visible erythema, edema, mortality, or overt behavioral abnormalities therefore provides information only on the short-term endpoints evaluated after this specific exposure. The experiment did not assess repeated-dose dermal toxicity, cumulative irritation, skin sensitization, dermal absorption, or systemic iron exposure. Because the release of free or exchangeable iron from the formulation and its penetration through the skin were not measured, no conclusion can be drawn regarding repeated exposure or the use of this system by individuals with disorders of iron metabolism.

The acute eye irritation test in New Zealand rabbits was conducted with reference to OECD 405. Fluorescein sodium staining and ocular examination showed no corneal opacity, iris abnormalities, conjunctival hyperemia, edema, or yellow-green fluorescence staining in the treated eyes at 24, 48, or 72 h after exposure (Figure 6d). The treated eyes were comparable to the self-control eyes, indicating no observable acute ocular irritation under the tested conditions [52]. These data are limited to the acute ocular endpoints and observation period used in this experiment and do not establish safety under repeated periocular exposure or long-term human use.

Overall, the HET-CAM, single-exposure dermal assessment, and acute eye irritation results showed no obvious irritation under the specific acute endpoints and test conditions evaluated in this study. These observations are descriptive and do not establish that the architecture of the deposited coating or a particular Fe^3+^–polyphenol coordination mechanism was responsible for the observed responses. They should not be interpreted as evidence of general biocompatibility, absence of toxicity, or safety during repeated human use [53].

The safety evaluation remains preliminary. It did not address long-term repeated exposure, cumulative irritation, skin sensitization, delayed contact allergy, genotoxicity, chronic or systemic toxicity, the safety of possible photodegradation products, or human scalp compatibility. Free-iron release, dermal penetration, and systemic exposure were not measured; consequently, no conclusion can be drawn regarding individuals with disorders of iron metabolism. Further assessment should follow a tiered, risk-based strategy, including characterization of releasable iron and dermal exposure, evaluation of skin-sensitization and genotoxicity endpoints, and, when supported by adequate nonclinical evidence and ethical approval, repeated-use and human compatibility studies. The present results support further investigation but are insufficient to establish complete or long-term safety.

### 2.6. Proof-of-Concept ResNet50-Based Image Evaluation and SHAP Analysis of the Reconstructed Colorimetric Score

Natural plant extracts are inherently affected by raw-material origin, harvest season, extraction conditions, and batch-to-batch variation, which may lead to fluctuations in dyeing performance. Conventional visual assessment of hair dyeing quality can be subjective and difficult to standardize, whereas instrumental colorimetry provides quantitative and reproducible color descriptors [54]. In the present study, instrumental colorimetry remained the primary method for evaluating dyeing performance. The deep-learning component was not required to establish the extraction, dyeing, or interfacial-coating conclusions. Instead, it was included as an exploratory analysis to examine whether standardized hair tress photographs could approximate a predefined score derived from colorimetric measurements and might therefore serve as a potential low-cost screening adjunct when instrumental measurements are unavailable. A ResNet50 model was used for image-based score estimation, while SHAP analysis was applied separately to examine the internal consistency of the colorimetric scoring framework [55,56,57].

**Figure 7:**
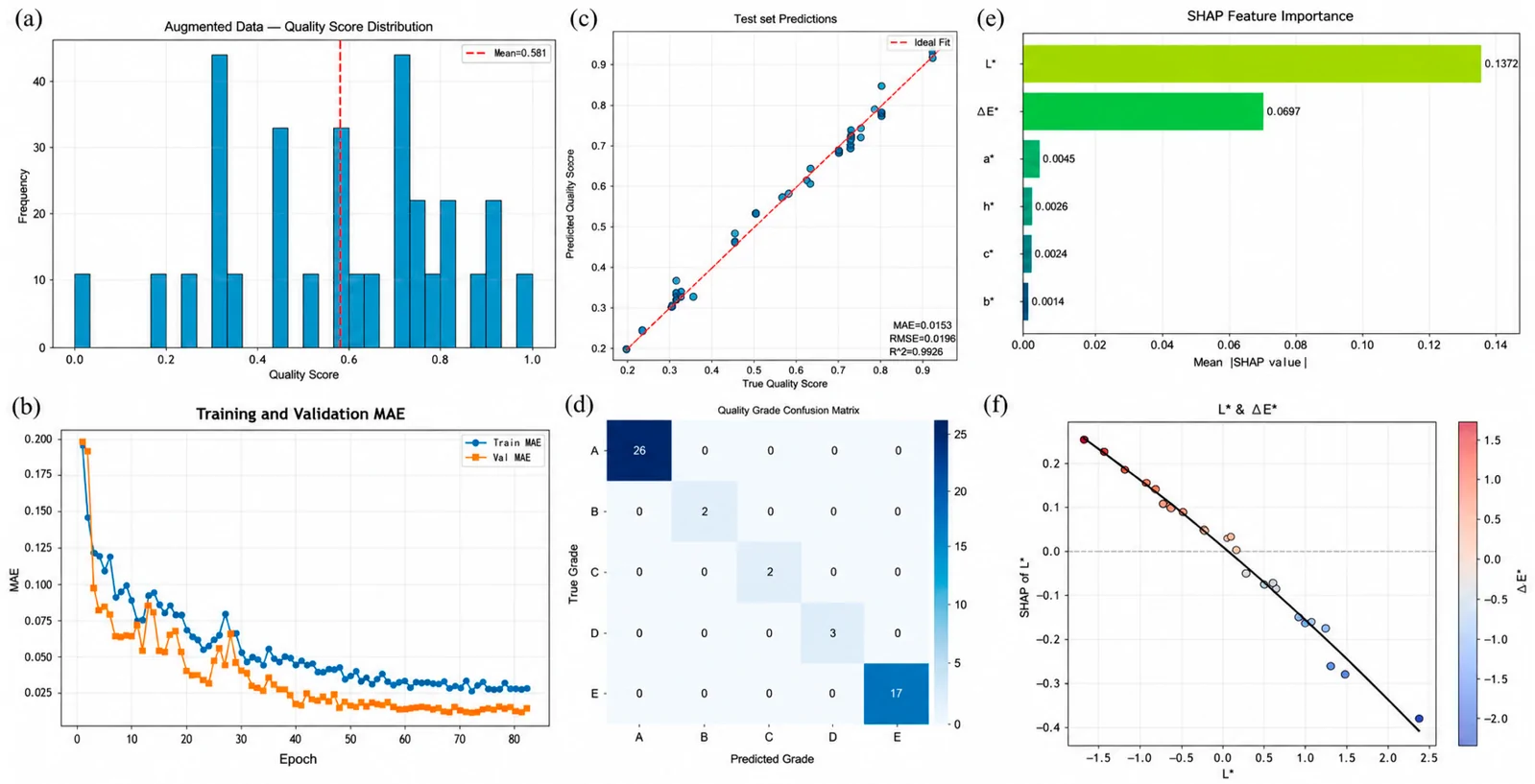
Internal proof−of−concept ResNet50 evaluation and separate SHAP analysis of the reconstructed colorimetric score. (**a**) Distribution of reconstructed scores after dynamic normalization. (**b**) Training and validation MAE curves under the current augmented-image split. (**c**) Predicted versus calculated scores in the internal test subset. The displayed metrics represent apparent internal performance and do not constitute external validation. (**d**) Confusion matrix for exploratory five-level classification within the current dataset. (**e**) SHAP-based global importance ranking of colorimetric variables used to analyze the reconstructed score; this analysis was not applied to pixel-level ResNet50 features. (**f**) SHAP dependence plot illustrating the mathematical relationship between L*, ΔE*, and the constructed score.

The original dataset contained 30 independent dyed hair tress samples, each consisting of one standardized image and the corresponding colorimetric parameters. Most original samples had L* values in the range of 20–50 and ΔE* values in the range of 35–60, indicating clear color development. To increase the number of image instances available for model fitting, data augmentation was performed using geometric transformations, including rotations of ±15° and ±30° and horizontal or vertical flipping; color-space perturbations, including independent ±20% adjustments in brightness, contrast, and saturation; and Gaussian noise injection [46,58]. The augmentation procedure generated 330 image instances from the 30 original samples. These augmented instances were transformations of the original images and did not represent additional independent experimental samples. They should therefore be regarded as an interpolation around the limited original-image distribution rather than as an expansion of the underlying biological, formulation, or hair-type diversity. In addition, because the target score was color-dependent, the use of brightness-, contrast-, and saturation-based transformations may have introduced some inconsistency between the transformed image appearance and the original colorimetric label. Accordingly, this analysis was treated as a proof-of-concept evaluation, and its generalizability across batches, processes, hair types, imaging devices, and independently acquired samples remains to be established. Reconstruction of the quality score was performed before model training. The original scoring formula used fixed denominators, which compressed the score range to approximately 0.8–0.95 and limited its ability to distinguish quality grades. Dynamic Min–Max normalization was therefore used to reconstruct the score:

M = 0.6 × (Lmax − L*)/(Lmax − Lmin) + 0.4 × (ΔE* − ΔEmin)/(ΔEmax − ΔEmin)

where Lmax, Lmin, ΔEmax, and ΔEmin were derived from the dataset. The weighting coefficients of 0.6 and 0.4 were assigned based on expert judgment regarding the relative importance of lightness and total color difference. After reconstruction, the score distribution occupied a larger proportion of the available numerical range, as shown in Figure 7a, with a peak in the intermediate range of approximately 0.5–0.6 and representation of both higher- and lower-score samples [59]. This reconstructed value should be regarded as a study-specific colorimetric index rather than as an independently validated expert, consumer, or commercial-quality standard. Its distribution and grade boundaries were dependent on the current dataset and scoring assumptions.

A ResNet50 network initialized with ImageNet-pretrained weights was selected as a conventional transfer-learning baseline [55,56,57]. ResNet50 was chosen because its residual architecture is well established, its pretrained implementation is readily reproducible, and transfer learning allows previously learned image features to be reused when only a limited number of original images are available. It was not selected through a systematic comparison with alternative architectures and is therefore not claimed to be the optimal network for this task. The input was a 224 × 224 × 3 RGB hair tress image. The convolutional backbone and global-average-pooling layer were retained, while the fully connected layer was replaced by a custom regression head with the architecture 2048 → 512 → 128 → 1, using ReLU activation, Dropout of 0.3, and a single output corresponding to the reconstructed score [60]. The 330 augmented image instances were divided into training, validation, and test subsets at a ratio of 7:1.5:1.5, corresponding to 230, 50, and 50 image instances, respectively. Because the documented split was performed at the augmented-image level, complete independence among transformed derivatives of the same original hair tress image was not demonstrated. The training and validation curves in Figure 7b therefore describe optimization behavior under the current internal split and should not be interpreted as evidence of performance on independent real samples. Training was performed using a ReduceLROnPlateau learning-rate scheduler with an initial learning rate of 1 × 10^−4^, a patience value of 5 epochs, and a decay factor of 0.5. Early stopping was applied by monitoring validation MAE with a patience value of 15 epochs. Training stopped at epoch 82, and the lowest validation MAE of 0.0122 occurred at epoch 72.

Under the current internal split, the regression model produced MAE = 0.0153, RMSE = 0.0196, and R^2^ = 0.9926 in the nominal test subset. In the predicted-versus-calculated scatter plot shown in Figure 7c, most data points were distributed close to the y = xy = xy = x line, with a maximum absolute deviation of 0.048. These results indicate close agreement with the reconstructed score within the present augmented-image distribution. However, these values should be interpreted as apparent internal proof-of-concept performance rather than as unbiased estimates of generalization. Because the number of independent original samples was only 30 and independence among augmented derivatives across the current subsets was not demonstrated, the reported metrics may be optimistic and cannot be extrapolated to independently acquired hair samples, different hair types, different formulations, or different imaging conditions.

For exploratory quality-grade classification, the reconstructed scores were divided into five grades: A ≥ 0.8, B = 0.6–0.8, C = 0.4–0.6, D = 0.2–0.4, and E < 0.2. Under the current internal split, the model produced an apparent classification accuracy of 100%, and the corresponding confusion matrix contained no off-diagonal elements (Figure 7d). This observation indicates that the model separated the constructed grades within the present augmented-image dataset. It should not, however, be interpreted as externally validated classification accuracy or as evidence that the model is ready for practical deployment. Independent original samples, particularly samples close to the predefined grade boundaries, are required to assess the robustness of this classification framework.

To examine the relationship between the reconstructed score and conventional colorimetric variables, SHAP analysis was performed using L*, a*, b*, C*, h°, and ΔE* [61,62,63,64]. This analysis was separate from the pixel-level ResNet50 model and did not explain the convolutional features or image regions used by the neural network. The global feature-importance ranking showed that L* and ΔE* were the dominant variables associated with the reconstructed score (Figure 7e), with their combined mean absolute SHAP contribution exceeding 95%. In contrast, a*, b*, C*, and h° each contributed only marginally. Because the scoring formula explicitly assigned weights of 0.6 and 0.4 to L* and ΔE*, respectively, their dominant SHAP contributions were mathematically expected. The SHAP result should therefore be interpreted as an internal consistency check of the constructed colorimetric score rather than as an independent discovery of the determinants of dyeing quality or as an explanation of the ResNet50 decision process.

The SHAP dependence analysis showed that lower L* values and higher ΔE* values were associated with higher reconstructed scores (Figure 7f). This pattern is consistent with the definition of the score and with the colorimetric interpretation that lower lightness corresponds to darker coloration, while greater ΔE* reflects a larger color difference relative to the original substrate [65]. The dependence plot therefore illustrates the mathematical relationship embedded in the scoring framework; it does not establish a newly learned biological or mechanistic interaction between L* and ΔE*.

Overall, the image-based component provides a preliminary demonstration that standardized hair tress images can approximate a study-specific colorimetric score within the current dataset. Its purpose was exploratory and supplementary to instrumental colorimetry, rather than essential to the principal chemical and materials conclusions of the study. ResNet50 was used as a conventional pretrained baseline and was not demonstrated to outperform simpler regression approaches, shallow convolutional networks, or lightweight pretrained architectures. Before practical application can be considered, future studies should increase the number of independent real samples, perform sample-level grouping before augmentation, restrict transformations that alter label-relevant color information, validate a locked model on an independently acquired external dataset, and compare ResNet50 with simpler and more computationally efficient baseline models. Accordingly, the present model is not proposed as a deployable tool or as a replacement for instrumental colorimetry or expert assessment.

## 3. Materials and Methods

### 3.1. Materials and Reagents

Fresh fruits of *Aronia melanocarpa* were supplied by Jilin Qiuzhiyuan Ecological Technology Co., Ltd. (Changchun, China). Cellulase (200 U/mg, Shandong Longkete Enzyme Preparation Co., Ltd., Dezhou, China) and pectinase (30 U/mg, Shandong Longkete Enzyme Preparation Co., Ltd., Dezhou, China) were used for polyphenol extraction. Ammonium ferric citrate (Aladdin Biochemical Technology Co., Ltd., Shanghai, China) was selected as the Fe^3+^ source for mordanting because of its aqueous compatibility and suitability for the mild processing conditions used in the hair dyeing system, and L-cysteine (Kangjian Food Biotechnology Co., Ltd., Quanzhou, China) was used as the active component for hair pretreatment. Other reagents, including absolute ethanol (Sinopharm Chemical Reagent Co., Ltd., Shanghai, China), xanthan gum (Shenzhen Chengda Biotechnology Co., Ltd., Shenzhen, China), hydroxymethyl cellulose (Tianjin Huasheng Chemical Reagent Co., Ltd., Tianjin, China), EDTA-2Na (Tianjin Beichen Fangzheng Reagent Factory, Tianjin, China), peppermint essential oil (Huangshan Tianmu Peppermint Pharmaceutical Co., Ltd., Huangshan, China), vitamin D (Shenzhen Chengda Biotechnology Co., Ltd., Shenzhen, China), and routine analytical-grade chemicals, were used as received unless otherwise specified. Commercially prepared 10-level bleached hair tresses were used as a standardized high-contrast substrate. The low-melanin background increased sensitivity to color deposition and reduced variation in the initial shade during formulation screening. Because bleaching alters cuticle morphology, porosity, lipid content, and keratin oxidation, this substrate does not represent normal unbleached hair, and the results should not be directly generalized to all hair types. The main instruments included an ultrasonic cleaner (KQ3200DE, 320 W; Kunshan Ultrasonic Instruments Co., Ltd., Kunshan, China), a refrigerated high-speed centrifuge (3–30 KS; Sigma Laborzentrifugen GmbH, Osterode am Harz, Germany), an LC–MS/MS system (API4000; AB SCIEX, Concord, ON, Canada), a colorimeter (WF30; Shenzhen Wave Optoelectronics Technology Co., Ltd., Shenzhen, China), an electronic single-fiber tensile tester (HC-1000N;Tenso-Lab 4, 2512E-F; Mesdan S.p.A., Puegnago del Garda, Italy), a scanning electron microscope (SU8600; Hitachi High-Tech Corporation, Tokyo, Japan), ATR–FTIR (Nicolet iS20; Thermo Fisher Scientific, Madison, WI, USA), Raman spectroscopy (LabRAM HR Evolution; HORIBA France SAS, Palaiseau, France), XRD (SmartLab; Rigaku Corporation, Tokyo, Japan), EDS (Ultim Max 65; Oxford Instruments, Abingdon, UK), DSC (Discovery DSC 250; TA Instruments, New Castle, DE, USA), and a water contact angle analyzer (OCA 15EC; DataPhysics Instruments GmbH, Filderstadt, Germany).

### 3.2. Methods

#### 3.2.1. Extraction and Total Phenolic Yield Determination of *Aronia melanocarpa*

The *A. melanocarpa* fruits were thawed at room temperature, washed, dried, and ground to obtain dried fruit powder before extraction. Briefly, 1.00 g of the dried fruit powder was mixed with an ethanol solution at the designed solid–liquid ratio, followed by the addition of cellulase and pectinase according to the dry fruit mass. Ultrasound-assisted compound enzymatic extraction was performed at 320 W. The extracted total phenolic yield was determined using the Folin–Ciocalteu assay and expressed as mg gallic acid equivalents per g of dried fruit starting material (mg GAE/g dry fruit). The denominator used in this calculation was the initial mass of dried fruit powder used for extraction, rather than the mass of the recovered extract. The calibration curve for gallic acid was y = 0.0104x + 0.0858, with an R^2^ value of 0.9993. The reaction mixture consisted of 1 mL of sample or standard solution, 5 mL of 10% Folin–Ciocalteu reagent, and 4 mL of 7.5% Na_2_CO_3_. After incubation in the dark for 60 min, the absorbance was measured at 760 nm. Based on single-factor experiments and response surface optimization, the final extraction conditions were as follows: cellulase-to-pectinase mass ratio of 5:5, total enzyme dosage of 3%, ethanol volume fraction of 70%, solid–liquid ratio of 1:50, ultrasonication temperature of 40 °C, and ultrasonication time of 60 min. After extraction, the mixture was centrifuged at 9000 r/min for 10 min. The supernatant was collected, and the extracted total phenolic yield was calculated relative to the initial mass of dried fruit powder and expressed as mg GAE/g dry fruit.

#### 3.2.2. Limited Targeted LC–MS/MS Analysis of Selected Non-Anthocyanin Phenolics

For limited targeted LC–MS/MS analysis, 0.2324 g of the optimized *A. melanocarpa* polyphenol extract was dissolved in 70% methanol and diluted to 5 mL. The solution was ultrasonicated for 30 min, filtered through a 0.22 μm membrane, and diluted tenfold when required to bring the analytical response within the calibration range.

Chromatographic separation was performed on an Agilent Poroshell 120 EC-C18 column (Agilent Technologies, Inc., Santa Clara, CA, USA) (2.7 μm, 3 × 50 mm). The mobile phase consisted of 0.5% formic acid in water and acetonitrile, with a flow rate of 0.6 mL/min, an injection volume of 10 μL, and a column temperature of 35 °C. Mass-spectrometric detection was conducted in multiple-reaction-monitoring mode at a spray voltage of 4.5–5.5 kV, a desolvation temperature of 500 °C, and an N_2_ flow rate of 1000 L/h.

The predefined panel included 12 non-anthocyanin reference analytes: quercetin, caffeic acid, rutin, procyanidin B1, procyanidin B2, epicatechin, ferulic acid, hyperoside, protocatechuic acid, kaempferol, chlorogenic acid, and vanillic acid. The panel covered selected procyanidins, flavan-3-ols, flavonols, flavonol glycosides, and phenolic acids and reflected the scope of the available targeted analytical method. It was not designed as an untargeted analysis or as a complete representation of the phenolic composition of *A. melanocarpa*.

Anthocyanins were not included in the original target panel, and the method was not validated for anthocyanin-specific identification or quantification. Calibration curves were generated for the 12 selected reference analytes; however, unless validated sample-specific concentrations are calculated and reported, these calibration data are used only to describe the analytical range of the target panel and not to infer quantitative composition or analyte dominance.

#### 3.2.3. Construction of the Polyphenol-Based Hair Dyeing System

The crude polyphenol extract was concentrated under reduced pressure to remove ethanol, redissolved in distilled water, and filtered to obtain the polyphenol working solution for dyeing. Based on process screening, a three-step dyeing system was established, consisting of L-cysteine pretreatment, Fe^3+^ mordanting, and polyphenol dyeing. L-cysteine served as the main active component in the pretreatment formulation, ammonium ferric citrate served as the Fe^3+^ source in the mordanting formulation, and *A. melanocarpa* polyphenol extract served as the core chromogenic component. After comprehensive evaluation of colorimetric parameters and hair fiber mechanical properties, the optimal process conditions were determined as follows: pretreatment at pH 9 with 8% L-cysteine at 45 °C for 40 min; mordanting at pH 4.5 with 8% ammonium ferric citrate at 45 °C for 40 min; and dyeing at pH 5.5 with a 20% (*w*/*v*) solution of the freeze-dried/crude *A. melanocarpa* polyphenol extract at 45 °C for 40 min.

#### 3.2.4. Dyeing Procedure and Color Evaluation

To compare the effects of different metal mordants and dyeing sequences on color development, ferrous sulfate, copper sulfate, magnesium sulfate, calcium chloride, zinc sulfate, and ammonium ferric citrate were used to provide Fe^2+^, Cu^2+^, Mg^2+^, Ca^2+^, Zn^2+^, and Fe^3+^, respectively. Four dyeing procedures were compared: direct dyeing, simultaneous mordanting and dyeing, dyeing followed by mordanting, and mordanting followed by dyeing. For each group, 2 g of bleached hair tresses was treated with the corresponding formulations according to the designed sequence. After treatment, the hair samples were rinsed with water and dried naturally or using forced air. Colorimetric parameters, including L*, a*, b*, C*, h*, and ΔE*, were measured using a WF30 colorimeter. Each sample was measured at least three times, and the mean value was used for analysis. Representative images of hair tresses were also recorded to compare color depth and uniformity. The tensile properties of hair fibers were measured using an HC-1000N electronic single-fiber tensile tester. The preload force was 1 N, and the stretching speed was 10 mm/min. Five replicate measurements were performed for each group.

#### 3.2.5. Washing Fastness and Light-Stability Tests

The environmental stability of the dyed hair samples was evaluated using washing and light-exposure tests. For washing fastness, dyed hair tresses were washed 15 times at 25, 35, 45, and 55 °C, with each washing cycle lasting 1 min. Color differences before and after washing were measured. Subsequently, additional washing cycles were performed at 45 °C for up to 50 cycles to record changes in ∆E* as a function of the number of washing cycles. For light stability, dyed hair tresses were exposed to UVA irradiation and simulated sunlight. The simulated sunlight intensity was set at 20,000 lux, and the total exposure time was 60 h. Samples were collected every 5 h and equilibrated at room temperature in the dark for 30 min before colorimetric measurement. Tensile strength and elongation at break after light exposure were measured to evaluate the mechanical stability of the dyed hair fibers under light-induced stress.

#### 3.2.6. Adsorption Kinetic and Equilibrium Measurements

To evaluate the uptake of the *A. melanocarpa* polyphenol extract by hair fibers, 1.00 g of hair was immersed in 40 mL of the optimized polyphenol solution and maintained at 25, 35, 45, or 55 °C under constant agitation. Aliquots of 0.5 mL were collected after 5, 10, 30, 60, 90, 120, 180, and 240 min. The polyphenol concentration remaining in solution was determined using the Folin–Ciocalteu method. The uptake at time t was calculated as follows:

Qt = (C0 − Ct)V/m

where C0 and Ct are the initial and time-dependent polyphenol concentrations, respectively; V is the solution volume; and m is the mass of the hair sample.

For equilibrium measurements, hair samples were equilibrated with polyphenol solutions of different initial concentrations for 3 h at the selected temperatures. The equilibrium uptake was calculated as follows:

Qe = (C0 − Ce)V/m

where Ce is the equilibrium polyphenol concentration. The experimental data were represented using the pseudo-second-order kinetic equation and the Langmuir and Freundlich equilibrium equations. These equations were used as empirical models and were not assumed to establish a unique adsorption mechanism.

#### 3.2.7. Interfacial and Structural Characterization of Hair Fibers

To characterize treatment-associated surface and structural changes and to evaluate whether these changes were consistent with interfacial Fe^3+^–polyphenol association, bleached undyed hair, polyphenol-treated hair, and polyphenol–Fe^3+^-treated hair were examined using complementary surface, spectroscopic, elemental, diffraction, and thermal analyses. Surface morphology was examined by SEM at an accelerating voltage of 3.0 kV after sputter-coating. Water contact angles were measured using a DSA25 contact angle analyzer (KRÜSS GmbH, Hamburg, Germany). ATR–FTIR spectra were collected over the range of 4000–500 cm^−1^ with a resolution of 4 cm^−1^ and 64 scans. Raman spectra were recorded using a 532 nm laser at a power of 5 mW, over the range of 400–3500 cm^−1^ with a resolution of 2 cm^−1^. XRD analysis was performed using Cu radiation at 40 kV and 40 mA, with a step size of 0.02° and a scanning range of 3–80°. The diffraction profiles were subjected to peak deconvolution using Origin (2020), and the resulting values are reported as calculated apparent relative crystallinity. Because the analysis was performed on whole treated hair samples, these values were treated as operational descriptors of the fitted diffraction profiles rather than as direct measurements of keratin molecular reorganization. EDS was used to analyze the elemental distribution of C, O, and Fe. For DSC analysis, dried whole-hair samples were cut into small pieces, and approximately 3 mg was heated from 20 to 300 °C at a rate of 10 °C/min. The resulting Td and ΔHd values were treated as whole-sample keratin-associated thermal transition parameters rather than as direct measures of molecular ordering.

#### 3.2.8. Preliminary Acute-Irritation Screening and Exposure Assessments

All animal-related procedures were reviewed and approved by the Animal Ethics and Welfare Committee of Tianjin Jinke Bona Biotechnology Co., Ltd., Tianjin, China. The mouse dermal exposure and irritation assessment was approved under Approval No. GENINK-20250081, and the rabbit eye irritation test was approved under Approval No. GENINK-20250079. Both approvals were issued on 18 July 2025, and the approved study period was from 22 July to 1 August 2025. The animal use facility was licensed under SYXK(Jinbin)2023-0005. The approved protocols involved six SPF female ICR mice aged 6–8 weeks for the dermal assessment and three male New Zealand white rabbits weighing 2–3 kg for the ocular irritation assessment. All procedures were conducted in accordance with institutional animal welfare requirements and the 3Rs. Animals were acclimatized before testing and monitored for local irritation signs, behavioral abnormalities, body-weight changes, and other signs of distress during the observation period.

To characterize acute irritation endpoints under the specified exposure conditions, HET-CAM screening, a single-exposure dermal assessment in mice, and an acute eye irritation assessment in rabbits were performed. For the dermal assessment, the dorsal skin of mice was shaved over an area of approximately 2 cm × 3 cm. Then, 1.5 g of the test dyeing formulation was uniformly applied to the exposed skin and covered with an occlusive dressing for 24 h. After removal of the residual formulation, erythema, edema, and behavioral changes were assessed, and body weight was measured at 0, 24, 48, and 72 h.

For the acute eye irritation test, 0.1 mL of the test liquid was instilled into the conjunctival sac of the right eye of New Zealand white rabbits. The eyelids were gently closed for 4 s, after which the eye was rinsed with physiological saline. The left eye served as the within-animal control. Corneal, iris, and conjunctival reactions were observed at 24, 48, and 72 h, with additional staining with 2% sodium fluorescein.

For the HET-CAM assay, fertilized eggs were incubated until day 10 and then windowed. Approximately 200 μL of the test sample was applied to the chorioallantoic membrane surface. Physiological saline (0.9%) was used as the negative control, and 0.01% SDS was used as the positive control. Hemorrhage, coagulation, and vascular lysis were recorded at 0, 5, and 60 min.

These experiments were designed to evaluate acute endpoints only. They did not assess repeated-dose toxicity, cumulative irritation, skin sensitization, genotoxicity, chronic toxicity, dermal iron absorption, systemic exposure, or human compatibility.

#### 3.2.9. Statistical Analysis

All experiments were performed at least in triplicate unless otherwise stated, and the results are expressed as mean ± standard deviation. Statistical analyses were conducted using appropriate statistical software. Differences among multiple groups were evaluated by one-way analysis of variance followed by Duncan’s multiple range test. A value of *p* < 0.05 was considered statistically significant. In addition to statistical significance testing, experimentally meaningful magnitude changes were reported using absolute differences, percentage changes, and relevant measured parameters where appropriate. Response surface optimization was performed using Design-Expert 13.0. (Stat-Ease Inc., Minneapolis, MN, USA), and the adequacy of the regression model was evaluated based on the analysis of variance, the coefficient of determination, and the lack-of-fit test. Other data processing, curve fitting, and plotting were performed using conventional statistical and graphing software.

## 4. Conclusions

This study evaluated a source-specific application of established Fe^3+^–polyphenol assembly chemistry using a compositionally complex *Aronia melanocarpa* extract. Ultrasound-assisted cellulase–pectinase extraction yielded 82.315 mg GAE/g dry fruit of extractable total phenolics under the optimized conditions. The LC–MS/MS experiment was limited to a predefined panel of 12 selected non-anthocyanin analytes and did not provide a comprehensive quantitative composition of the extract. The available calibration data did not establish the abundance or quantitative dominance of these analytes, and the anthocyanin fraction was not analyzed. Therefore, the present results do not identify a single dominant phenolic component or Fe^3+^ ligand and do not resolve the contribution of pH-sensitive anthocyanins to color development or interfacial assembly.

A sequential L-cysteine pretreatment–Fe^3+^ mordanting–polyphenol dyeing protocol produced a color difference in ΔE* = 55.42 while retaining the measured hair fiber mechanical performance under the tested conditions. The polyphenol extract was applied only during the final dyeing stage at pH 5.5, after the pH 9 pretreatment and pH 4.5 mordanting formulations had been separately applied and rinsed from the hair. This sequence avoided direct exposure of the extract to the alkaline pretreatment formulation. Nevertheless, pH- and Fe^3+^-dependent transformations of the anthocyanin fraction were not directly monitored, and their effects on color development, stability, and metal association remain unresolved.

The separation of pretreatment, mordanting, and dyeing provided experimental control over cuticle conditioning, Fe^3+^ deposition, and subsequent phenolic application. However, the optimized protocol involved three separate 40 min treatment stages with intermediate rinsing and was therefore more time-consuming and operationally complex than the single-application format used by many commercial hair dye products. No representative commercial oxidative or plant-derived hair dye was included as a matched control. Consequently, the present study does not establish equivalence or superiority in processing time, color range, color uniformity, washing fastness, light stability, hair damage, irritation potential, or overall consumer convenience relative to commercial products.

Complementary SEM, water contact angle, ATR–FTIR, Raman, EDS, XRD, and DSC analyses were consistent with the presence of a relatively uniform Fe-containing polyphenol coating at the hair fiber interface. These results support interfacial Fe^3+^–polyphenol association involving multiple phenolic ligands, but they do not resolve the exact ligand composition, Fe^3+^ coordination number, ligand-to-metal stoichiometry, or three-dimensional coordination geometry. The XRD profiles showed changes consistent with increased calculated apparent relative crystallinity, whereas DSC showed only modest changes in whole-sample thermal transition behavior. Neither result demonstrates molecular reorganization, reconstruction, or restoration of keratin.

No obvious acute irritation was observed in the HET-CAM, single-exposure dermal assessment, or acute eye irritation assay under the tested conditions. These findings are limited to the specific acute endpoints evaluated and do not establish safety under repeated human use, cumulative irritation, skin sensitization, genotoxicity, chronic or systemic exposure, or exposure to possible photodegradation products. Because free-iron release, dermal penetration, and systemic exposure were not measured, the present study also provides no basis for a safety conclusion regarding individuals with disorders of iron metabolism.

Overall, the principal contribution of this work is not the introduction of Fe^3+^–polyphenol MPN chemistry as a new materials concept. Instead, the study extends established coordination-assembly principles to an astringency-associated *A. melanocarpa* extract and integrates phenolic recovery, sequential hair fiber treatment, and application-oriented performance assessment. Further development should focus on monitoring anthocyanin speciation and color changes under relevant pH and Fe^3+^ conditions, simplifying the current three-step protocol into a one- or two-application format, and conducting matched comparisons with representative commercial oxidative and plant-derived hair dyes on both bleached and unbleached hair. Comprehensive compositional characterization should include validated sample-specific quantification of the selected non-anthocyanin analytes, dedicated profiling of the anthocyanin fraction, and broader untargeted analysis to determine the relative contributions of different ligand classes.

## Figures and Tables

**Table 1:** Analytical calibration information for the 12 selected non-anthocyanin analytes included in the limited targeted LC–MS/MS panel.

NO.	Compound	Linear Regression Equation	R^2^	Linear Range (ppm)
1	Quercetin	y = 1480x − 324,000	0.9992	0~1
2	Caffeic acid	y = 4860x − 1,520,000	0.9991	0~0.5
3	Rutin	y = 1400x + 175,000	0.995	0~10
4	Procyanidin B1	y = 307x + 72,000	0.992	0~0.5
5	Procyanidin B2	y = 307x + 72,000	0.992	0~0.5
6	Epicatechin	y = 56.8x + 12,000	0.995	0~0.5
7	Ferulic acid	y = 10.1x + 4700	0.994	0~0.5
8	Hyperoside	y = 332x + 72,000	0.997	0~5
9	Protocatechuic acid	y = 3370x + 1,920,000	0.985	0~5
10	Kaempferol	y = 378x + 602,000	0.978	0~0.5
11	Chlorogenic acid	y = 1260x + 450,000	0.991	0~5
12	Vanillic acid	y = 462x − 102,000	0.997	0~0.5

Note: This table reports calibration parameters for the selected reference analytes only and does not report their concentrations in the *A. melanocarpa* extract. The data cannot be used to infer quantitative dominance or percentage contribution to total phenolics. Anthocyanins were not included in this targeted analytical panel.

**Table 2:** CIELAB color parameters of hair fibers dyed with *Aronia melanocarpa* polyphenols using different metal mordants.

Metal Ion	L*	a*	b*	c*	h*	∆E*
Fe^3+^	30.22	−0.85	−6.08	6.14	262.05	53.08
Fe^2+^	46.88	8.93	3.21	9.49	19.76	35.76
Cu^2+^	40.45	10.57	4.90	12.75	27.09	41.91
Mg^2+^	53.56	9.86	5.35	11.22	28.48	29.41
Ca^2+^	71.34	4.3	9.34	5.28	65.30	10.53
Zn^2+^	73.78	1.19	4.50	4.66	75.15	9.99

## Data Availability

The datasets used or analyzed during the current study are available from the corresponding author on reasonable request.

## Supplementary Materials

The following supporting information can be downloaded at: https://www.mdpi.com/article/10.3390/plants15162500/s1, Table S1: Experimental design and responses of the Box–Behnken design (BBD) for ultrasound-assisted compound enzymatic extraction of polyphenols from *Aronia melanocarpa*; Table S2: Analysis of variance (ANOVA) and regression model statistics for the Box–Behnken response surface model; Figure S1: Single-factor experiments and response surface analysis for optimization of ultrasound-assisted compound enzymatic extraction of polyphenols from *Aronia melanocarpa*. (a) Cellulase-to-pectinase ratio; (b) total enzyme dosage; (c) solid–liquid ratio; (d) ethanol volume fraction × solid–liquid ratio; (e) solid–liquid ratio × ultrasonic extraction temperature; (f) solid–liquid ratio × ultrasonic extraction time; (g) ultrasonic extraction temperature × ultrasonic extraction time. Data are presented as Mean ± SD, n = 3; Table S3: Colorimetric parameters of hair samples obtained using different dyeing sequences in the Fe^3+^-mordanted system; Figure S2: Complete single-factor optimization of softening, dyeing, and mordanting conditions. (a–d) Pretreatment conditions, including pretreatment agent concentration, pretreatment pH, pretreatment time, and pretreatment temperature; (e–h) dyeing conditions, including dyeing pH, polyphenol concentration, dyeing time, and dyeing temperature; (i–l) mordanting conditions, including mordanting pH, mordant concentration, mordanting time, and mordanting temperature. In each panel, the bar chart represents elongation at break, and the line chart represents tensile strength. Data are presented as mean ± SD, n = 3. Different lowercase letters indicate significant differences among groups (*p* < 0.05, Duncan’s test); Table S4: Parameters obtained from the linearized pseudo-second-order representation of *Aronia melanocarpa* polyphenol uptake by hair fibers at different temperatures; Figure S3: Fitting analysis of the mechanical properties of dyed hair and the adsorption behavior of polyphenols on hair fibers. (a) Changes in elongation at break of dyed hair over time; (b) adsorption kinetic curves of polyphenols on hair fibers at different temperatures; (c) pseudo-second-order kinetic fitting curves for polyphenol adsorption on hair fibers at different temperatures; (d) adsorption isotherm of polyphenols on hair fibers; (e) Freundlich adsorption isotherm fitting plot. Data are presented as mean ± SD, n = 3; Table S5: Parameters obtained from the linearized Langmuir and Freundlich representations of polyphenol uptake by hair fibers at different temperatures; Table S6: Calculated apparent relative crystallinity of differently treated hair samples estimated from XRD peak deconvolution; Table S7: Whole-sample keratin-associated thermal-transition parameters of differently treated hair samples determined by DSC.
