# Supplementary material for: Fe3+-Mediated Interfacial Polyphenol Coatings on Hair Fibers Using an Aronia melanocarpa Extract
Source: Plants (Basel). 2026 Aug 18;15(16):2500. doi: 10.3390/plants15162500 (PMC13516680; doi:10.3390/plants15162500)
Supplement: Supplementary file 1 [file plants-15-02500-s001.zip › plants-4460124-supplementary.pdf]

Table S1. Experimental design and responses of the Box–Behnken design (BBD) for ultrasound-assisted compound enzymatic extraction of polyphenols from *Aronia melanocarpa.*

NO.	Factors				Polyphenol Yield（mg/g）
	A	B	C	D	
1	60	50	50	40	62.6442
2	70	60	60	30	66.1538
3	70	50	50	50	62.3019
4	80	60	60	40	69.5692
5	70	60	50	40	64.3115
6	70	40	60	30	58.8154
7	80	50	50	40	65.9404
8	70	40	60	50	55.3154
9	60	50	70	40	71.5577
10	70	50	70	50	58.4885
11	60	40	60	40	64.2385
12	70	40	50	40	55.6615
13	60	60	60	40	72.3346
14	70	50	70	30	60.0385
15	70	40	70	40	56.7769
16	70	50	60	40	80.1923
17	70	50	60	40	80.9519
18	70	60	70	40	66.1077
19	70	50	50	30	61.6827
20	60	50	60	30	62.2827
21	80	50	70	40	55.8846
22	70	50	60	40	81.3846
23	70	60	60	50	68.3769
24	70	50	60	40	80.9135
25	80	50	60	30	69.2712
26	80	50	60	50	56.4038
27	80	40	60	40	58.4923
28	70	50	60	40	81.8977
29	60	50	60	50	76.5173

Table S2. Analysis of variance (ANOVA) and regression model statistics for the Box–Behnken response surface model.

Source	Sum of Squares	df	Mean Square	F Value	*p*-Value	Significance
Model	2091.86	14	149.42	133.03	< 0.0001	**
A	96.41	1	96.41	85.83	< 0.0001	**
B	276.04	1	276.04	245.75	< 0.0001	**
C	1.13	1	1.13	1.01	0.3321	
D	0.0589	1	0.0589	0.0524	0.8222	
AB	2.22	1	2.22	1.98	0.1814	AB
AC	89.96	1	89.96	80.09	< 0.0001	**
AD	183.63	1	183.63	163.48	< 0.0001	**
BC	0.1159	1	0.1159	0.1032	0.7528	
BD	8.19	1	8.19	7.29	0.0173	
CD	1.18	1	1.18	1.05	0.3235	
A^2	210.05	1	210.05	187.01	< 0.0001	**
B^2	562.64	1	562.64	500.91	< 0.0001	**

C^2	807.59	1	807.59	719	< 0.0001	**
D^2	570.3	1	570.3	507.73	< 0.0001	**
Residual	15.73	14	1.12	—	—	
Lack of fit	14.13	10	1.41	3.55	0.1168	
Pure error	1.59	4	0.3982	—	—	
Total	2107.58	28	—	—	—	

Note: * $p < 0.05$; ** $p < 0.01$.

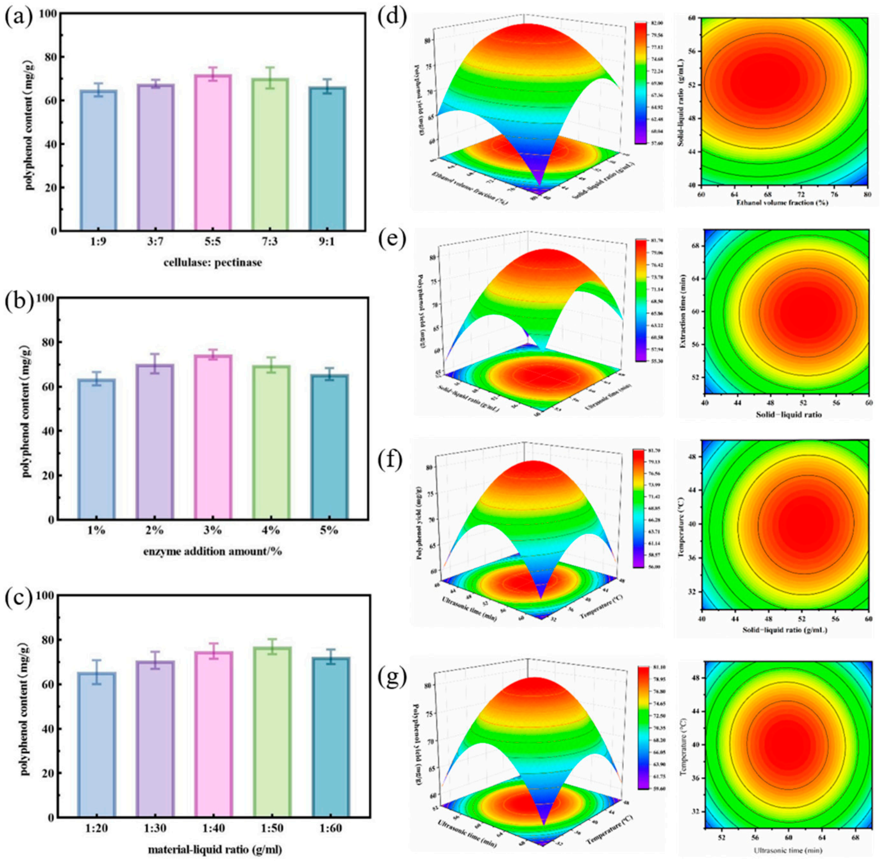

Figure S1. Single-factor experiments and response surface analysis for optimization of ultrasound-assisted compound enzymatic extraction of polyphenols from *Aronia melanocarpa*. (a) Cellulase-to-pectinase ratio; (b) total enzyme dosage; (c) solid–liquid ratio; (d) ethanol volume fraction × solid–liquid ratio; (e) solid–liquid ratio × ultrasonic extraction temperature; (f) solid–liquid ratio × ultrasonic extraction time; (g) ultrasonic extraction temperature × ultrasonic extraction time. Data are presented as Mean ± SD, n = 3.

Table S3. Colorimetric parameters of hair samples obtained using different dyeing sequences in the Fe^{3+}-mordanted system.

Dyeing Sequence	L*	a*	b*	c*	h*	ΔE*
Direct Dyeing	81.68	0.37	12.63	12.64	88.33	2.07
Simultaneous Mordanting and Dyeing	45.09	2.84	13.97	14.25	138.52	35.32
Dyeing Followed by Mordanting	35.57	-0.94	4.83	4.92	198.97	45.15
Mordanting Followed by Dyeing	26.31	-1.12	-1.19	1.63	233.20	55.42

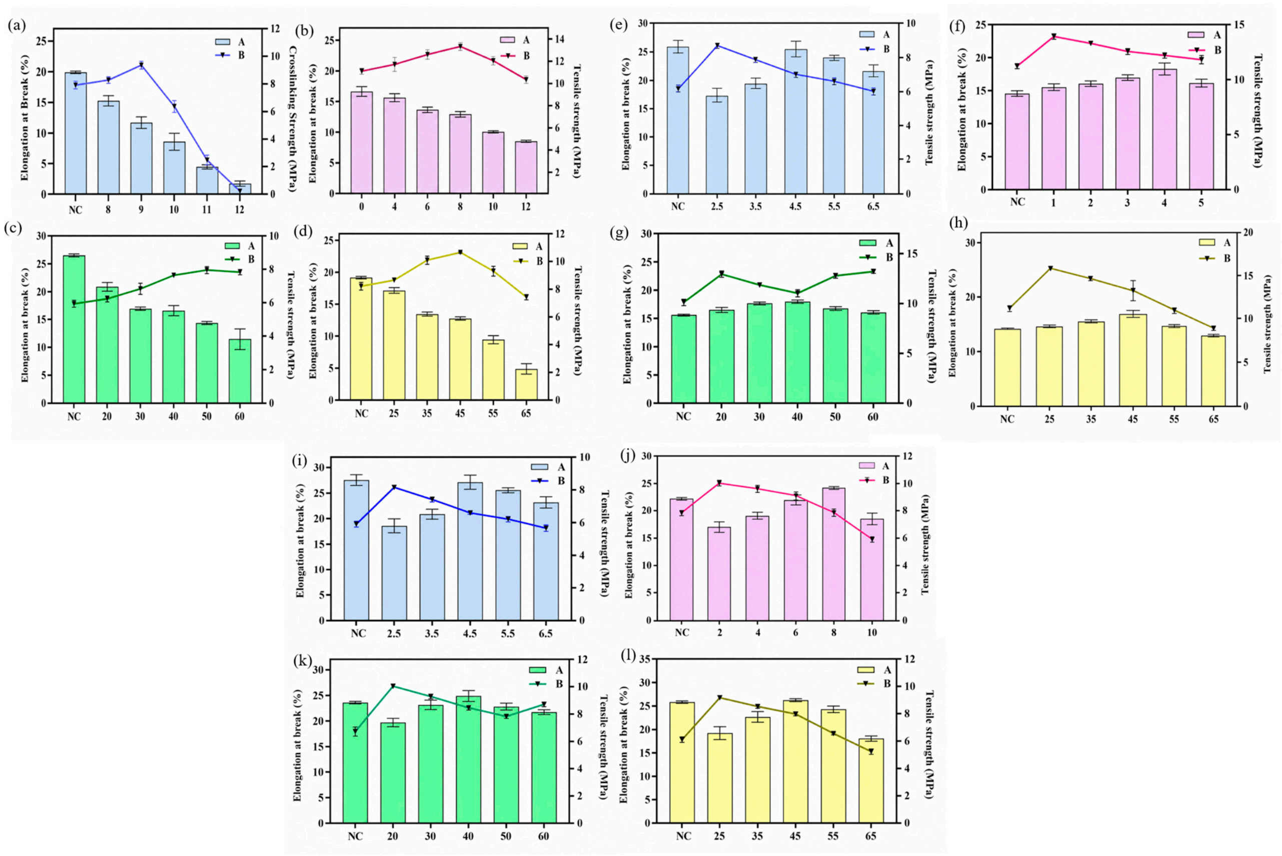

Figure S2. Complete single-factor optimization of softening, dyeing, and mordanting conditions. (a–d) Pretreatment conditions, including pretreatment agent concentration, pretreatment pH, pretreatment time, and pretreatment temperature; (e–h) dyeing conditions, including dyeing pH, polyphenol concentration, dyeing time, and dyeing temperature; (i–l) mordanting conditions, including mordanting pH, mordant concentration, mordanting time, and mordanting temperature. In each panel, the bar chart represents elongation at break, and the line chart represents tensile strength. Data are presented as mean ± SD, n = 3. Different lowercase letters indicate significant differences among groups ($p < 0.05$, Duncan's test).

Table S4. Parameters obtained from the linearized pseudo-second-order representation of *Aronia melanocarpa* polyphenol uptake by hair fibers at different temperatures.

Temperature (℃)	Regression Equation	K_2 [g/(mg·min)]	Qe,2 (mg/g)	R^2
25	t/Qt=1.1855t+47.7493	0.0294	0.8434	0.9641
35	t/Qt=0.9543t+34.8970	0.0261	1.0478	0.9809
45	t/Qt=0.6754t+23.8818	0.0191	1.4805	0.9677
55	t/Qt=0.56536t+10.5802	0.0302	1.7687	0.9915

Note: K_2 and Qe,2 are parameters obtained from the linearized pseudo-second-order equation, and R^2 describes the agreement of this representation with the experimental data. A high R^2 value does not establish chemisorption, identify a unique rate-limiting step, or determine the molecular interactions responsible for polyphenol uptake.

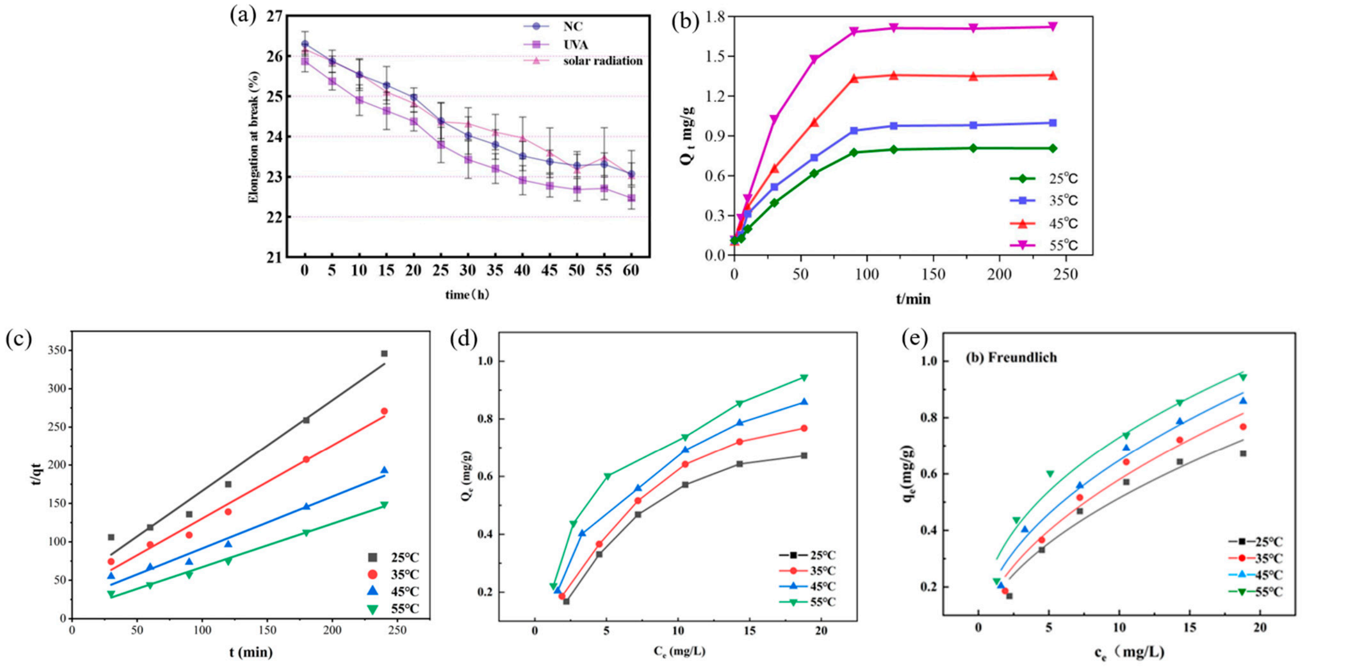

Figure S3. Fitting analysis of the mechanical properties of dyed hair and the adsorption behavior of polyphenols on hair fibers. (a) Changes in elongation at break of dyed hair over time; (b) adsorption kinetic curves of polyphenols on hair fibers at different temperatures; (c) pseudo-second-order kinetic fitting curves for polyphenol adsorption on hair fibers at different temperatures; (d) adsorption isotherm of polyphenols on hair fibers; (e) Freundlich adsorption isotherm fitting plot. Data are presented as mean ± SD, n = 3.

Table S5. Parameters obtained from the linearized Langmuir and Freundlich representations of polyphenol uptake by hair fibers at different temperatures.

Temperature (°C)	Langmuir			Freundlich		
	Qm（mg/g）	KL（L/mg）	R2	KF（L/mg）	1/n	R2
25	1.04704	0.10588	0.985	0.14981	0.53574	0.937
35	1.18686	0.10469	0.991	0.16756	0.53927	0.958
45	1.17838	0.13773	0.994	0.20547	0.49903	0.976
55	1.15335	0.2043	0.984	0.26578	0.43849	0.964

Note: Qm and KL are parameters of the Langmuir representation, whereas KF and 1/n are parameters of the Freundlich representation. The higher R^2 values obtained for the Langmuir equation indicate a closer empirical fit within the tested concentration range but do not demonstrate a completely homogeneous surface, actual monolayer coverage, or a unique adsorption mechanism.

Table S6. Calculated apparent relative crystallinity of differently treated hair samples estimated from XRD peak deconvolution.

Hair Sample Type	Crystallinity (%)
Uncolored hair (bleached only)	28.88 ± 0.32
Polyphenol–Fe^{3+} dyed hair	37.36 ± 0.76
Polyphenol-dyed hair	30.48 ± 0.34

Note: The reported values were calculated from peak-deconvoluted XRD profiles of whole treated hair samples. They are analysis-dependent and may include contributions from keratin-associated scattering, the deposited coating, background selection, sample packing or orientation, and the deconvolution procedure. The values should not be interpreted as direct evidence of keratin molecular reorganization or restoration.

Table S7. Whole-sample keratin-associated thermal-transition parameters of differently treated hair samples determined by DSC.

Sample	Td (℃)	ΔHd (J/g)
Uncolored hair	232.4	10.77
Dyeing hair	234.1	10.49
Iron-dyeing hair	234.3	10.83

Note: Td denotes the observed keratin-associated denaturation peak temperature, and ΔHd denotes the corresponding mass-normalized enthalpy of the whole treated hair sample. These parameters describe whole-sample thermal-transition behavior and should not be interpreted as direct measures of keratin crystallinity, molecular ordering, or structural restoration. The differences should be interpreted cautiously because replicate variability was not reported and the deposited coating may contribute to the total sample mass and thermal response.
